# Engineered live bacteria as disease detection and diagnosis tools

**DOI:** 10.1186/s13036-023-00379-z

**Published:** 2023-10-24

**Authors:** Imen Tanniche, Bahareh Behkam

**Affiliations:** 1 Department of Mechanical Engineering, Virginia Tech, Blacksburg, VA 24061 USA; 2 School of Biomedical Engineered and Sciences, Virginia Tech, Blacksburg, VA 24061 USA; 3 Center for Engineered Health, Institute for Critical Technology and Applied Science, Virginia Tech, Blacksburg, VA 24061 USA

**Keywords:** Whole-cell biosensors, Living machines, Biomedical diagnostics, Imaging vectors, Synthetic biology

## Abstract

Sensitive and minimally invasive medical diagnostics are essential to the early detection of diseases, monitoring their progression and response to treatment. Engineered bacteria as live sensors are being developed as a new class of biosensors for sensitive, robust, noninvasive, and in situ detection of disease onset at low cost. Akin to microrobotic systems, a combination of simple genetic rules, basic logic gates, and complex synthetic bioengineering principles are used to program bacterial vectors as living machines for detecting biomarkers of diseases, some of which cannot be detected with other sensing technologies. Bacterial whole-cell biosensors (BWCBs) can have wide-ranging functions from detection only, to detection and recording, to closed-loop detection-regulated treatment. In this review article, we first summarize the unique benefits of bacteria as living sensors. We then describe the different bacteria-based diagnosis approaches and provide examples of diagnosing various diseases and disorders. We also discuss the use of bacteria as imaging vectors for disease detection and image-guided surgery. We conclude by highlighting current challenges and opportunities for further exploration toward clinical translation of these bacteria-based systems.

## Introduction

Diagnostics are a crucial aspect of clinical practice for identifying the presence of diseases, monitoring their progression, and designing efficacious treatments; as such, they are crucial in evaluating and improving global health. Many current state-of-the-art detection and diagnostic tools have limited sensitivity for early disease detection or require several days for a conclusive diagnosis. Moreover, most conventional methods are often complex and require costly equipment; therefore, they cannot be implemented in resource-limited settings [1–3]. To overcome these limitations, there is a mounting interest in developing more sensitive, accurate, rapid, sustainable, and cost-effective detection and diagnostic tools [2, 3].

Recent advances in synthetic biology and disease etiology have provided new opportunities for developing living sensors capable of more sensitive detection and more accurate diagnosis [4]. Given the diversity and abundance of bacteria in the human body (*i.e.*, estimated at 10:1 to 1:1 bacteria/human cell ratio [5, 6]) and their simple and easily manipulatable genome, these microorganisms provide a unique opportunity as cost-effective, sustainable, dynamic, and rapid biosensing vectors for medical applications. Leveraging significant advances in genetic engineering, bacterial whole-cell biosensors (BWCBs) are constructed to function as living machines with seamlessly integrated sensing, processing, and communication units to respond to the presence of analytes and produce detectable output signals [4, 7]. The use of BWCBs as a platform for in vivo and in vitro medical diagnostics is advantageous for multiple reasons. Bacteria are easy to handle, have a low cost of production, can proliferate in relatively inexpensive media, and can recycle themselves. Self-replicating bacteria can express detectable signals and recognition elements such as fluorescent proteins, biomarkers, and antibodies; thus, eliminating the need for expensive purification steps. Whole-cell biosensors have highly specific recognition sites that detect target analytes directly from the biospecimens or in vivo without complex sample preparation or the need for analytical instruments [8–10]. Other significant advantages of the BWCBs are sensitivity, specificity, and robustness to detect physiologically-relevant concentrations of biomolecules of interest in complex environment with high signal-to-noise ratio [7, 11]. Furthermore, bacteria can survive harsh environments (*e.g.*, low pH, hypoxia, and high temperature) and preserve the functionality of the sensor components. In addition, BWCBs can be deployed in currently inaccessible in vivo environments to detect labile, rapidly metabolized, or inactivated molecules for unprecedented clinical testing capabilities [12, 13]. Another advantage of BWCBs is their engineerability and versatility. Utilizing synthetic biology tools, complex and sophisticated circuits with innovative functionalities for robust diagnosis of various diseases have been developed. All these qualities make BWCBs development an attractive avenue for next-generation detection of biomolecules of interest in human health and disease onset [10, 14, 15].

BWCBs have brought forth new biomolecular diagnostic capabilities (*e.g*., identification of the first biological thiosulfate sensor [13]), genetic repurposing of existing biological parts (*e.g*., heme sensor for gastrointestinal bleeding [16]), creation of rapid dynamic sensors [12, 13, 17, 18], and quantitative point of care testing [9, 19, 20]. Certain BWCBs have enabled highly sensitive in vitro disease detection in a fraction of the time of conventional tests [18, 20, 21]. In recent years, more interest has been directed toward BWCBs for early detection in vivo. BWCBs have also found new utility in interrogating host-response in situ and noninvasively to advance our understanding of disease development and progression. The new generation of BWCBs offers unprecedented capabilities, such as dynamic sensing with memory for near real-time monitoring of disease progression [12, 15, 22] and closed-loop detection regulated-treatment [23].

In this review, we first summarize recent examples of engineered bacterial biosensors for disease detection and diagnosis. We will emphasize examples of gastrointestinal disorders, cancers, infectious diseases, and metabolic disorders. Next, we focus on the use of BWCBs and bacterial cells as imaging vectors in target tissues or organs for early disease detection or image-guided surgery. Finally, we offer our perspective on challenges and opportunities in developing bacterial-based diagnostics.

## Bacterial-based disease detection

**Fig. 1:**
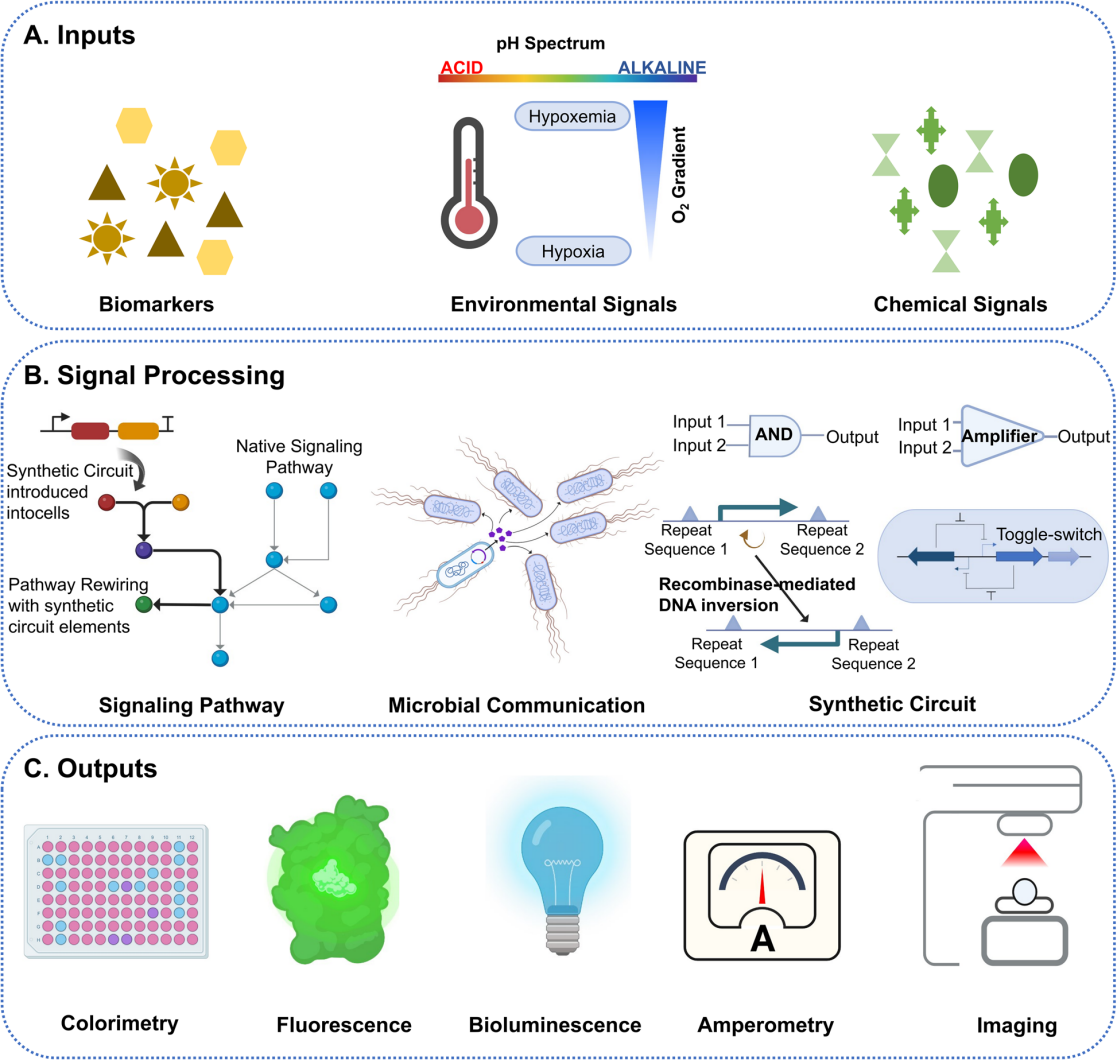
Components of Bacterial whole-cell biosensors (BWCBs) as diagnostic tools. **A** BWCBs receive inputs in the form of biomarkers, chemical signals (including host-produced metabolites and synthetic compounds), and environmental signals (including temperature, pH, and oxygen level). **B** They process input signal(s) using rewired signaling pathways, engineered cell–cell signaling circuits for population-scale sensing, and synthetic sensing circuits. **C** Finally, BWCBs produce reporter outputs such as colorimetric, fluorescent, or bioluminescent signals, current, or medical imaging (e.g., CT) contrast agents

Detecting relevant disease markers at the clinical point of care may facilitate disease prevention, early detection, and timely treatment. Bacterial-based disease detection is an emerging field offering a simple, rapid, noninvasive, and cost-effective alternative to assess analytes of interest. This class of bacterial sensors is engineered to detect and respond to biomolecules that are indicators of health or diseases (Fig. 1A) either in vivo or in clinical samples *ex vivo*. In this section, we describe these biotechnologies and examples of their applications.

### Bacterial Whole-cell Biosensors (BWCBs)

BWCBs are genetically engineered living bacteria that detect analytes with high sensitivity and specificity and report this information. Whole-cell sensing can be performed through three different approaches (Fig. 1B): (i) rewiring the existing signaling pathways within the cells [24], (ii) harnessing cell–cell signaling synthetic circuits for coordinated population-scale sensing (*i.e.*, quorum sensing) [25, 26], and (iii) introducing synthetic sensing circuits. Synthetic circuits are assemblies of different genetic parts and regulatory modules to regulate gene expression in response to various external stimuli. The simplest and most conventional form of genetic circuits is the inducible system [27], which uses a specific transcription factor. The DNA binding strength of this transcription factor depends on its target ligand [27]. Bacteria use a variety of mechanisms to sense their environment and can integrate multiple signals. Therefore, complex genetic circuits with different logic gates have been built from the simple AND gate to the complex NOR gate [28]. An AND gate integrates two inputs, and the output is only ON when all inputs are present [29]. A growing number of genetic logic circuits (*e.g.*, OR, NAND, XOR, XNOR) are being constructed [28, 30, 31] to equip BWCBs with higher-order sensing capabilities. Moreover, memory circuits have been developed to allow bacteria to remember previous exposure to disease biomarkers. Currently, memory circuits are based on transcription factors-based toggle switches [12, 22] and recombinases [32]. The typical output of BWCBs are reporter proteins that produce colorimetric, fluorescent, or bioluminescent signals, current, or medical imaging contrast agents (Fig. 1C).

**Fig. 2:**
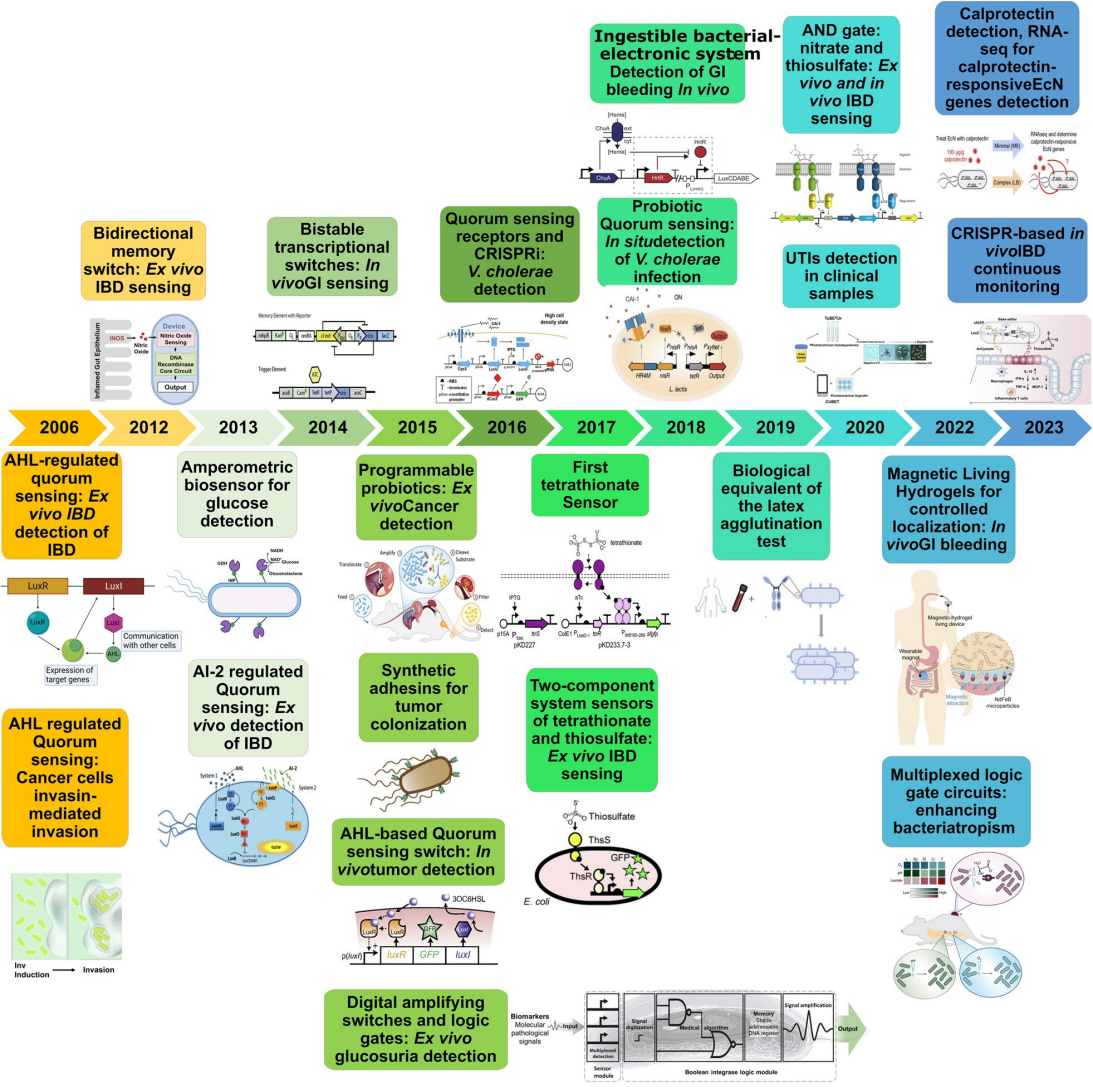
Summary of progress in BWBCs for the detection of diseases and disorders. In 2006, an AHL biosensor was developed for ex vivo detection of inflammatory bowel disease (IBD). Representation of AHL-dependent regulation of quorum sensing in LuxR/LuxI-type systems from [33]. Design of induction-dependent invasion of a cancer cell in 2006. Schematic of the activation of biosensor at a critical bacterial cell density or in a hypoxic environment resulting in the synthesis of invasin and the bacterial invasion of tumor cells [25]. In 2012, *E. coli* was engineered to detect and respond to gut inflammation in mouse ileum explants through nitric oxide sensing. Representation of biosensor where sensing nitric oxide results in switching of the core circuit and causes a permanent change in gene expression output [32]. In 2013, the first the use of the GDH-bacteria for sensitive amperometric glucose biosensor was reported. Schematic of the construction of genetically engineered bacteria displaying GDH and INP. GDH catalyzes the transformation of glucose to gluconolactone in the presence of coenzyme NAD + , which is reduced to NADH [34]. Development of BWCB based on the detection of quorum sensing molecule AI-2 for the quantitative detection of IBD in saliva, stool, and intestinal samples of IBD patients. Representation of the hypothesized mechanism of quorum sensing-regulated bioluminescence in *V. harveyi* [24]. In 2014, the development of bistable transcriptional switches for in vivo detection of gut inflammation through ATC. Schematic of the memory circuit. Representation of the lambda cI/Cro-based transcriptional memory and the tetP-Cro trigger elements [22]. In 2015, probiotics were engineered for detection of cancer in urine [10]. Programming controlled adhesion of *E. coli* to target tumors with synthetic adhesins [35]. Development of quorum sensing switch in *Salmonella* for in vivo detection of tumors. Schematic of *lux* quorum sensing system. [26] Detection of biomarkers in human clinical samples via amplifying genetic switches and logic gates. Schematic architecture of the biosensor. A sensor module enables multiplexed detection of pathological biomarkers. These control signals induce a Boolean integrase logic gate module. Boolean integrase logic gates enable signal digitization, amplification, and storage of the diagnosis test’s outcome in a stable DNA register [9]. In 2016, development of BWCB based on *V. cholerae* quorum sensing receptors and CRISPRi for cholera diagnosis [18]. In 2017, development of the first tetrathionate sensor. Establishment of two-component systems of tetrathionate and thiosulfate for the detection of IBD in fecal and colon samples. Schematic of thiosulfate sensor and the schematic of ligand-induced signaling for thiosulfate sensor characterization [13]. In 2018, development of Ingestible Micro-Bio-Electronic Device (IMBED) for gastrointestinal bleeding sensing in porcine model. Schematic of the blood sensor gene circuit [16]. Development of BWCB for cholera sensing and reporting by engineered *L. lactis *in vitro and in vivo. Engineered CAI-1–dependent signaling in *L. lactis* [36]. In 2019, development of the biological equivalent of the latex agglutination test. Representation of cell agglutination using a BWCB surface-displaying nanobodies which bind selectively to a target protein analyte [19]. In 2020, development of AND logic gate with nitrate and thiosulfate for ex vivo sensing of IBD. Schematic of AND logic gate that uses nitrate and thiosulfate genetic circuits [14]. Development of BWCB bioluminescence-based assay for the diagnosis of urinary tract infection. Schematic representation of TuBETUr and CUBET diagnosis platforms [20]. In 2022, use of magnetic living hydrogels for in vivo detection of gastrointestinal bleeding. Schematic of the mechanism of the magnetic living hydrogels localized and retained in the intestine [37]. Development of multiplexed biosensors-based logic gate circuits for bacterial tropism enhancement. Schematic of biosensors for specific oxygen, lactate and pH levels detection to enable bacteria tropism in vivo [17]. In 2023, the development of intelligent responsive bacteria for diagnosis and therapy (i-ROBOT) for in vivo IBD monitoring. Schematic of i-ROBOT for thiosulfate sensing. Fluorescence and inheritable signals (genomic molecular recording and colorimetric output) enable IBD detection and monitoring [23]. A biosensor was developed for the detection of calprotectin, the gold standard biomarker of gut inflammation, with sensitivity and specificity in IBD patients. Schematic of the identification of calprotectin-responsive genes in *E. coli* Nissle 1917 using RNA-sequencing method [21]

Recent advances in molecular and synthetic biology have oriented the development of cell-based biosensors toward clinical applications, including disease detection, diagnosis, and monitoring of treatment outcomes. A timeline graph summarizing progress in BWCBs for disease detection is presented in Fig. 2. BWCBs are envisioned to be employed for sensing and reporting human diseases in vivo or in clinical samples ex vivo. BWCBs have been developed to diagnose diseases linked to the microbiome, including gastrointestinal disorders and urinary tract infections (UTIs). BWCBs are also finding use in the detection of cancers and other infections, each of which is discussed below.

#### Sensing of gastrointestinal disorders

The inaccessibility and complexity of the gut combined with the highly localized and temporally dynamic nature of many gut diseases result in diagnosis and therapy difficulties. Symptoms of many gut conditions are similar [38] and prone to change due to temporal changes in the microbiota composition of individual patients over time and differences in the microbiota composition across the patient populations (*e.g*., host genetics, age, diet, medication, and physical activity) [39–41]. These variations further complicate disease diagnosis. Standard diagnostic procedures such as endoscopy and colonoscopy provide only a snapshot of spatial information and are invasive and costly [42]. An alternative standard diagnostic method, stool sampling, is noninvasive but lacks spatial and temporal information. Furthermore, many gut inflammatory responses go undetected in stool sampling because the chemical species involved in the response are highly localized, transient, or degrade before excretion. Gut bacteria have highly advanced sensing machinery for gut cues and the ability to sense and record instantly biological and environmental factors in situ for the detection of localized and transient cues. Bistable transcriptional switches comprise memory circuits that can sense and remember transient environmental stimuli [43] transmitted through a trigger element or one of the transcription factors in the switch [44, 45]. Artificial gene-based memory systems use these transcriptional switches as sophisticated reporters that record biological events. Such systems have great potential for detecting, recording, and reporting transient biomarkers in the gut environment. These types of memory circuits are suitable for developing stable biosensors for in vivo diagnostics. For instance, in a proof of concept study, the well-characterized cI-Cro lysis-lysogeny switch of λ phage was integrated into a synthetic circuit enabling a real-time response to the presence of orally administered anhydrotetracycline (ATC) in the mouse gut [22]. A two-part system was constructed in *Escherichia coli* with a “trigger element” in which the lambda Cro gene is transcribed from an ATC-inducible promoter and a “memory element” derived from the cI/Cro region of λ phage. After being orally administered to mice, *E. coli* was recovered from fecal samples to evaluate the fraction of bacteria in the Cro state, measured by β-galactosidase reporter gene fused to Cro. The established memory was maintained for several days in the mouse gut context. Furthermore, this genetic memory circuit was used in conjunction with multiple biosensor triggers to identify gut inflammation cues [15]. This system was employed in a subsequent study to detect tetrathionate [12]. Although tetrathionate is transiently formed during intestinal inflammation [46], tetrathionate-responsive *E. coli* NGF-1 was capable of specifically sensing it. These studies show the benefits of engineered bacteria as noninvasive reporters of transient events in the body. The bacterial memory device showed ‘memory’ upon tetrathionate exposure by expressing Cro (*i.e.*, trigger) and β-galactosidase from the synthetic memory element and maintaining their expression in the absence of tetrathionate. The tetrathionate sensor was demonstrated to detect gut inflammation in both *Salmonella* Typhimurium-induced colitis model and the genetically engineered mouse model of chronic inflammatory bowel disease (IBD) and retain a memory of exposure over six months [12].

**Fig. 3:**
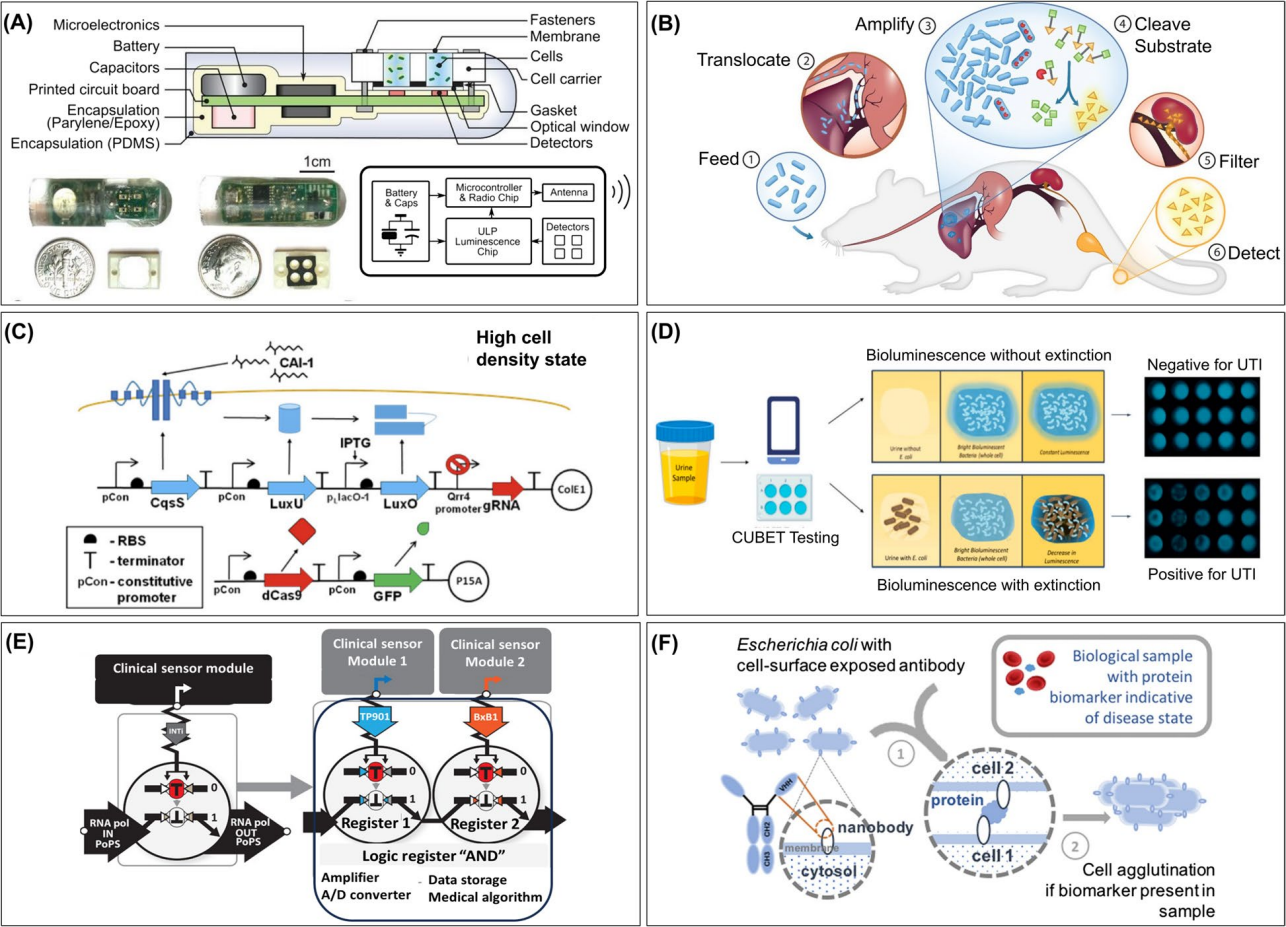
Representative bacterial whole cell biosensors (BWCBs). **A** Design and in vitro and in situ evaluation of Ingestible Micro-Bio-Electronic Device (IMBED) to detect gastrointestinal bleeding. Reprinted with permission from [16] **B** Selective colonization of bacteria in cancerous tissue harnessed for detecting liver metastases. The probiotic *E. coli* Nissle 1917 was engineered to express β-galactosidase enzyme LacZ to metabolize systemically injected LuGal into luciferin, detectable in urine. Reprinted with permission from [10]. **C** Design of a *Vibrio cholerae* detection system in *E. coli*. The quorum sensing proteins CqsS, LuxU and LuxO from *V. cholera* were combined with a genetic inverter (CRISPR) for tight control of GFP expression upon detecting *V. cholera.* At high cell density, CAI-1 dephosphorylate CqsS which dephosphorylate LuxO. Dephosphorylated LuxO cannot activate Qrr4 promoter and subsequently gRNA is not expressed. In the absence of gRNA, GFP is expressed. Reprinted with permission from [18]. **D** Detection of bacteria-based urinary tract infections (UTIs) using cellphone-based UTI bioluminescence extinction technology (CUBET). Reprinted with permission from [20]. **E** Architecture and functional composition of transcriptor-based digital amplifying genetic switches. The clinical sensor promoter drives integrase expression; integrase inverts a transcriptor module that controls the flow of RNA polymerase (RNA pol) along the DNA. Two transcriptors that respond to different signals can be composed in a series to produce an AND gate. A/D, analog to digital. Reprinted with permission from [9]. **F** Protein biomarker test for point-of-care medical diagnostics. Engineered *E. coli* cells with surface-displayed nanobodies are mixed with a biological sample. If the sample contains the protein biomarker indicative of a diseased state, cross-linking between bacterial cells and biomarker molecules occurs, leading to cell agglutination and change of the test sample's appearance. Reprinted with permission from [19]

BWCBs have also been integrated with electronic signal transduction circuitry. For instance, Ingestible Micro-Bio-Electronic Device (IMBED) integrates a probiotic bacterial sensor with ultra-low-power microelectronics (Fig. 3A) [16]. As a proof-of-concept, IMBED was developed to detect gastrointestinal bleeding in a porcine model via heme liberated from lysed blood cells. The engineered *E. coli* Nissle 1917 responded to increasing blood level (input) with luminescence output. Bioluminescence from activated *E. coli* cells was detected by phototransistors and transmitted as photocurrent data. IMBEDs’ sensitivity was 83.3% at 60 min, and specificity was 100% at 120 min. The IMBED platform has been adapted for sensing other disease-relevant small molecules, *e.g.*, thiosulfate and N-acyl homoserine lactone (AHL), as biomarkers of gut inflammation and an indication of bacterial infection, respectively [16].

Localized delivery is an important consideration for the clinical translation of BWCBs. Liu et al*.* incorporated genetically engineered bioluminescence-expressing heme-sensing bacteria into an ingestible protective magnetic hydrogel [37] to enable magnetically-controlled selective positioning of the bacterial biosensors within the different gut regions. X-ray microtomography was used to visualize the 3D spatial position of the magnetic hydrogels, and a wearable magnet attached to the abdominal skin was used to position it. The soft magnetic hydrogel conforms to the shape of the intestinal tract and decreases the risk of tissue damage. To assess the blood-sensing ability of the magnetic hydrogels in mice, gastrointestinal bleeding was induced by oral indomethacin administration. The magnets were removed after 12 h, and fecal samples were collected for luminescence analysis. Results revealed that the device functioned to detect gastrointestinal bleeding in vivo 24 h post-induction. Therefore, engineered bacteria encapsulated in the magnetic hydrogel could perform diagnosis at localized sites in the gut.

Inflammatory bowel disease (IBD) is a group of incurable disorders affecting the gastrointestinal tract [47]. Ulcerative colitis and Crohn’s disease represent the two main types of IBDs [48]. IBD patients experience flare-ups after a period of remission, requiring immediate detection and treatment of inflammation to stop disease progression. Current diagnosis and monitoring methods involve capsule endoscopy, blood test, computed tomography (CT), and magnetic resonance imaging (MRI). Additionally, colon biopsies can be taken to confirm the diagnosis [48]. There are limitations associated with each of these diagnostic procedures. Capsule endoscopy requires ingestion of the capsule, which might become impacted or trapped within the narrow areas in the small intestine. Furthermore, its motion through the gastrointestinal tract is not controlled; thus, critical functionalities such as adjusting the camera orientation or halting the capsule motion for more careful inspection of regions of interest are unfeasible. CT involves the ingestion of barium sulfate, which has potential adverse side effects. Moreover, radiation exposure is a significant limitation of CT, particularly in patients undergoing repeated examinations [49]. The main limitations of MRI are the long scan time and cost [50]. Finally, despite the efficacy of colon biopsies, there is a risk of injuries or punctures. Alternative noninvasive, easy, and rapid analysis methods can be very beneficial.

Noninvasive BWCB-based approaches were developed for ex vivo use with IBD patients' stool, saliva, and intestinal biopsy samples. For instance, a BWCB was developed and optimized for the detection of the bacteria interspecies signaling molecule autoinducer-2 (AI-2) [24]. The sensing system was based on *Vibrio harveyi* BB170, a variant genetically modified to only respond to AI-2. AI-2 is a quorum-sensing signaling molecule used for interspecies communication by Gram-positive and Gram-negative bacteria [51]. AI-2 was detected at considerably higher levels in all IBD patient samples compared to the samples from the control population. The method employs AI-2 as recognition elements and the *luxCDABE* operon as a bioluminescent reporter. These results suggest potential perturbations of the microflora in the inflamed intestine. Other studies have shown that bacteria producing AHL signaling molecules are suspected to be involved in several illnesses of gastrointestinal tract [52–54]. AHLs are used for communication between bacteria of the same species [55]. Consequently, sensors targeting AHLs have been developed for the selective and rapid detection of AHLs in saliva and stool samples using bioluminescence signal [33]. Comparison between human subjects with and without GI disorders shows the presence of AHLs in both groups but at different levels. Although both AI-2 and AHLs are broadly used for communication between bacteria, the existing works showed that the output of the sensors were not affected by crosstalk by either the bacterial sensors or the non-infectious microbiota [24, 33]. These BWCBS are sensitive, cost-effective, and have high throughput compared to current diagnostic methods.

Colonic thiosulfate and tetrathionate levels have been correlated with inflammatory conditions. Two-component system sensors of tetrathionate and thiosulfate were used as bacterial diagnostics with fluorescent protein output [13]. A novel genetically encoded thiosulfate sensor (ThsSR) and an improved tetrathionate sensor (TtrSR) from *Shewanella* species were characterized and employed to drive the expression of the superfolder GFP (sfGFP) in *E. coli* Nissle 1917. Briefly, the thiosulfate sensor contains ThsS, a membrane-bound sensor histidine kinase (SK) that phosphorylates the cytoplasmic response regulator (RR) ThsR in the presence of thiosulfate. The phosphorylated RR activates the transcription of the thiosulfate reductase operon, via the *sfgfp* promoter (P_*phsA342*_). Similarly, the tetrathionate sensor comprises the histidine kinase TtrS, that phosphorylates the cytoplasmic response regulator TtrR in the presence of tetrathionate. The phosphorylated TtrR activates transcription of the tetrathionate reductase operon, via the optimized *sfgfp* promoter (P_*ttrB185-269*_). The sensors were evaluated in the dextran sodium sulfate (DSS)-stimulated mouse model of colitis. Results showed that only the thiosulfate sensor activated the expression of the fluorescent protein reporter when exposed to the inflamed mouse gut. Although tetrathionate has previously been shown to accumulate during colonic infection [46], lack of detection could be because tetrathionate levels are not increased in the mouse model of colitis or because of the rapid consumption of tetrathionate by the microbiota at the mucosal site of production. If the latter is the case, genetic engineering could be used to increase sensors’ gain to detect lower tetrathionate levels.

Gut inflammation induces the expression of nitric oxide synthase and therefore increases the production of nitric oxide [56]. Using a similar two-component system sensor design, Woo et al. [14] built a nitrate-responsive genetic circuit using the NarX-NarL two-component system in the probiotic *E. coli* Nissle 1917. This sensor could sense high nitrate levels for colitis diagnosis and report it as a green fluorescent signal in the colon and fecal samples of the DSS-induced colitis mouse model. Moreover, a Boolean AND gate was implemented for simultaneous monitoring of thiosulfate and nitrate gut biomarkers to increase the sensor’s specificity for diagnosing gut inflammation.

Recombinase-based circuits were investigated since they enable the detection of transient input signals and induce a permanent DNA recombination. An engineered strain of *E. coli* EA3020 was constructed to detect IBD through nitric oxide sensing with the expression of cyan fluorescent protein (CFP) [32]. A bidirectional memory switch was constructed based on the *E. coli* fimbriae (Fim) phase variation system. The switch constitutively expresses yellow fluorescent protein (YFP) when the promoter *fimS* is oriented toward the Inverted Repeat Right (IRR), corresponding to the OFF state. Upon inversion (activation) of the switch, *fimS* is oriented toward the Inverted Repeat Left (IRL) (*i.e*., ON state), followed by the constitutive expression of CFP. The inversion of *fimS* is performed by the DNA recombinase FimE, which binds to the inverted repeat sequences. In this study, the bacterial enhancer-binding protein NorR was selected for its specificity to only nitric oxide [57], which binds to the *PnorV* promoter. The transcription of *fimE* was placed under the control of *PnorV*, which allowed the combination of nitric oxide sensing with the DNA recombinase core circuit. The engineered strain was co-cultured with mouse ileum explants. The detection of nitric oxide was accompanied by a permanent DNA recombination that is inherited after cell division.

Recently, the molecular specificity of BWCBs was leveraged to build a recombinase-based memory circuits in the probiotic *E. coli* E. cloni 10G to report transient biomarker panels, *i.e.,* nitric oxide, hydrogen peroxide (H_2_O_2_), tetrathionate, and thiosulfate by producing luminescence. These BWCBs were integrated in a miniaturized IMBED capsule for real-time detection of these biomarkers of IBD in porcine models of gastrointestinal inflammation [58].

Many gastrointestinal conditions cause elevated fecal calprotectin concentrations [59]. Currently, calprotectin is used as the clinical laboratory gold standard for IBD prognosis and diagnosis; however, results can take 1–2 weeks to become available [60]. The probiotic *E. coli* Nissle 1917 was engineered to detect calprotectin in murine models of IBD and stool samples from human patients with active IBD [21]. For this purpose, calprotectin-responsive genes were identified via RNA sequencing, their respective promoters were cloned upstream of a GFP reporter and screened for sensitivity and specificity. The optimized promoter was coupled with luxCDABE cassette to detect IBD in vivo within 7 days and in clinical stool samples within 12 h.

A promising opportunity for continuous monitoring and closed-loop detection-regulated treatment via BWCB was recently developed [23]. Intelligent responsive bacteria for diagnosis and therapy (i-ROBOT) were engineered to monitor and record IBD occurrence and progression noninvasively in real time and release treatment in DSS-induced mouse colitis. The three functions (*i.e.*, fluorescence reporting, base-editing system, and drug secretion) were simultaneously integrated into *E. coli* Nissle 1917. The i-ROBOT strains can sense inflammatory biomarkers (*i.e*., thiosulfate) and produce fluorescence. In addition, the response to biomarker stimulation is translated into permanent alterations in genomic DNA. The information is inherited in subsequent generations and results in the activation of functional protein expression (*i.e*., LacZ, where the activation of LacZ reports single-base conversion). Fluctuations in thiosulfate also induce the tunable release of the immunomodulator AvCystatin. Oral administration of i-ROBOT in mice generated real-time fluorescence and inheritable signals (*i.e*., genomic molecular recording and colorimetric output) in fecal and colon samples and attenuated inflammation. This BWCB enabled the simultaneous diagnosis and treatment in situ. The i-ROBOT platform is promising for continuously monitoring gastrointestinal and other metabolic disorders. The release of drugs in a self-modulated manner enables timely treatment of patients.

#### Cancer detection

Cancer is a leading cause of death worldwide [61]. Early detection provides the best chance for disease management and improved survival. Typically, physical exam, imaging, laboratory test, and biopsy are used to screen and diagnose cancer. However, some organs cannot be easily examined to determine the presence of a tumor or its grade. Certain imaging techniques, such as fluorescence imaging, have limited tissue penetration depth due to tissue absorption [62]. Another disadvantage of the clinical imaging modalities is limited sensitivity (*i.e.*, detection of positive results in patients with the illness) and inability to detect malignant masses smaller than 6–8 mm in diameter [63, 64]. Tissue biopsies are invasive and sometimes unsuccessful or unfeasible due to the inaccessibility of the tumor. Moreover, incisional biopsy presents a dissemination risk, which may cause implantation metastases [65]. Tumor biomarkers have also been utilized for screening. However, as many of the biomarkers are present in both normal and cancerous tissue, differential analysis to detect cancer may not be sensitive enough for early detection of malignancy or its recurrence [66].

In recent years, a growing number of studies have reported important advances in using bacteria for tumor therapies [67–70]. Several species of bacteria have been shown to colonize primary and metastatic tumors selectively. Furthermore, synthetic biology has enabled the design of targeted delivery systems that can sense tumor-specific biomarkers (for recent reviews, see [71, 72]). These findings can be used toward engineering genetic circuits for highly specific bacteria-based cancer detection.

A study exploited specific binding between bacterial outer-membrane proteins and disease-specific host molecules to concentrate the bacteria at the disease site, wherein these molecules are enriched. Synthetic adhesins (SAs) were constitutively expressed in an *E. coli* strain, allowing specific adherence and colonization of solid tumors in an ovarian cancer xenograft model [35]. A bioluminescent reporter was constitutively expressed to provide a sensitive readout of the bacterial adhesion process. The *E. coli* chassis used in this study is a mutant strain lacking a set of natural adhesins that have the potential to bind to receptors expressed on the surface of multiple host cells and tissues. Therefore, the expression of SAs in this strain promoted the selective adhesion to target tumor tissues and decreased the adhesion to non-target organs. The expression levels of the SAs and bioluminescence signal were identical in the inoculum and the tumor-recovered bacteria, indicating the stability of the expression of SAs in vivo. Furthermore, it was demonstrated that the bacterial dose needed for optimal tumor colonization in vivo was reduced by two orders of magnitude in SA-expressing *E. coli* compared to the wild type. In addition, titers of the engineered *E. coli* strain in the liver and spleen were lower than those of the wild-type *E. coli* strain (∼10–30 times lower in the spleen and ∼2–fourfold lower in the liver).

Oral administration of engineered probiotic *E. coli* Nissle 1917 strain resulted in preferential growth within mice metastatic liver tumors and expression of high levels of lacZ enzyme [10]. LacZ cleaved the systemically injected substrate (LuGal) and generated a high-contrast bioluminescence signal in the urine (Fig. 3B). Infiltrative hepatocellular carcinoma is difficult to detect with conventional imaging because of poor tumor-to-organ contrast. This approach could be useful for detecting primary hepatocellular carcinoma in patients at risk for malignant transformation.

Selective bacterial accumulation in the tumor microenvironment can be harnessed to develop BWCBs that target cancerous tissue. *E. coli* was engineered to use their cell density as a decision point for the invasion of cancer cells [25]. A genetic circuit was constructed in *E. coli* in which the *Vibrio fischeri* LuxI/LuxR mediated quorum sensing system regulated the *Yersinia pestis* invasin gene. The LuxI catalyzes the synthesis of AI-1 (*N*-3-oxohexanoyl-l-homoserine lactone), which accumulates in the media as the cell density increases. At high densities, AI-1 activates LuxR, which induces the expression of any gene incorporated into the operon [73], in this case, *inv* from *Y. pestis*. The quorum-sensing regulated invasin expression enabled the colonization of HeLa cells at a high cell density of *E. coli*. In another study, *S.* Typhimurium VNP200010 transformed with the LuxI/LuxR mediated quorum sensing system initiated green fluorescent protein (GFP) specifically in high-density colonies within tumors [26]. *Salmonella* was intravenously injected into subcutaneous mammary tumor-bearing mice. Selective colonization of *Salmonella* in tumors led to tumor-specific GFP expression and enabled visualization of the spatial distribution of bacteria at densities above 4.2 × 10^10^ CFU/g. Moreover, GFP expression was localized and was not produced when the average distance of neighboring colonies was greater than 155 µm. Such tumor-sensitive gene expression switch has a great potential to identify tumor cells specifically and express protein therapeutics in tumor tissue without damaging normal tissues.

Other strategies to improve tumor specificity of bacteria are to leverage genetic circuits that couple bacterial growth to the common physiological indicators (*i.e.*, oxygen, pH, or lactate) of tumors. For instance, Chien et al*.* combined synthetic circuits using AND logic gates to improve tumor specificity, reduce instances of bacteria mutation, and enable long-term biocontainment [17]. This study constructed a system by tuning and multiplexing bacterial growth with biosensors’ response to distinct environmental signatures in the mouse gastrointestinal tract and tumors. More specifically, promoter-biosensor machinery was coupled with bacterial growth in *E. coli* Nissle 1917 through the expression of essential genes. Results show that amplification through replication reached more than a 1,000-fold difference in the bacteria number using three independent biosensors. To test whether multiplexed containment circuits can enhance specificity in tumors, the combined lactate-hypoxia AND logic gate in *S.* Typhimurium ELH1301 was tested in the murine colon carcinoma model. The engineered strain was able to colonize tumors to a similar level as the control strain. But notably, the engineered bacteria number in the spleen and liver significantly decreased to 10^2^–10^3^ CFU/g, compared with the control strain's 10^4^–10^5^ CFU/g bacterial load. This system improves the sensitivity of BWCBs in the case of the insufficient or high basal expression of the reporter proteins.

#### Biosensing of bacterial infections

Infectious diseases include some of the most dreaded plagues and remain one of the leading causes of death worldwide [74]. The continual emergence of zoonotic diseases and antibiotic resistance highlights the importance of early detection and timely management of bacterial infections to prevent global pandemics and hinder the emergence of more antibiotic-resistant strains. Currently, specific diagnosis of bacterial infection requires isolation of the infectious pathogen in culture, microscopic visualization in tissue lesions, and/or detection of the host immune response to the microorganism. In certain cases, lumbar punctures, biopsies, or imaging scans are also utilized. Most of these procedures are either time-consuming or costly. They can also be invasive [75]. New detection and diagnostic technologies, such as BWCBs, can potentially revolutionize how laboratories identify the agents of bacterial diseases.

Cholera is a highly virulent and potentially lethal infectious disease caused by *Vibrio cholerae* bacteria. Cholera requires immediate treatment to replace lost fluids and electrolytes, because the disease can cause death 12–24 h post infection. Therefore, rapid diagnosis is critical. Unfortunately, available detection methods for cholera still have limitations. Conventional culture methods require trained technicians and laboratory infrastructure. Moreover, they are not precise and can take up to 8 days to confirm a cholera case [76]. Immunoassays that detect cholera toxins [77] or lipopolysaccharide [78] directly in stools and PCR-based analysis [79] are expensive and may be unfeasible in geographic regions with high cholera prevalence. Thus, a rapid and cost-effective strategy for the early detection of *V.* *cholerae* is of great interest. For this purpose, an *E.coli*-based BWCB that utilizes *V.* *cholerae* quorum sensing receptors and CRISPRi technology was developed [18]. *V. cholera* controls its infection cycle via a parallel quorum sensing system. There are two well-characterized pathways, both using two-component sensor proteins, CqsS and LuxPQ, which sense CAI-1 and AI-2 signaling molecules, respectively [80, 81]. The CAI-1 based system is used for intraspecies communications and is based on three proteins CqsS, LuxU and LuxO. These proteins were used as sensors in a synthetic circuit with a CRISPRi-based genetic inverter and a GFP reporter (Fig. 3C). The genetic inverter was used to control the expression of GFP. It prevented the expression of the GFP signal in the absence of CAI-1 sensor proteins. But when the CAI-1 sensor proteins are present, CRISPRi activity is blocked, allowing the expression of GFP. The sensor showed high sensitivity to the presence of *V.* *cholerae* supernatant with a tight control of expression of GFP signal. The biosensor utilizes part of the *V.* *cholerae* quorum sensing system (*i.e.*, CqsS), which has been shown to be very specific to CAI-1 [80], a molecule unique to the *V.* *cholerae* species. In contrast to most *V.* *cholerae* diagnostic tests that show either low sensitivity and/or specificity [82, 83], this sensor design provides a basis for future development of a highly sensitive, specific, and easy-to-use whole cell biosensor platform for the detection of *V. cholera*.

 Another study used the probiotic strain *Lactococcus lactis* for both detection and suppression of cholera in mice [36]. *L. lactis* was genetically engineered to specifically detect CAI-1 and express the fluorescent reporter protein mCherry. This BWCB showed 60-fold higher mCherry expression level in presence of CAI-1-producing bacteria compared to the control. Moreover, the detection circuit did not respond to environmental *Vibrio* strains (*e.g.*, *V. ordalii* or *V. lentus*) or to other non-CAI-1-producing bacteria. As a proof of concept of a living diagnostic tool with an easy point-of-care readout, mCherry was replaced by β-lactamase reporter whose activity is instantly visualized by a colorimetric test. The assay was tested in fecal pellets of infant mouse model. Only the fecal samples of cholera-infected mice treated with *L. lactis* showed positive signals after overnight incubation. These results indicate that in situ biosensing and reporting of *V. cholera* can be achieved with stable synthetic circuits and reporters.

*Pseudomonas aeruginosa* is a human pathogen that initiates respiratory infections by translocation from the gut or contamination of the airway [84]. *P. aeruginosa* is also responsible for increased mortality in gut-derived sepsis and bacteremia [85, 86], and it is also the most commonly detected Gram-negative bacteria in ulcers and burns [87, 88]. *E. coli* Nissle 1917 was engineered to detect AHL from *P. aeruginosa* and respond by releasing an anti-*P. aeruginosa* toxin in vitro [89]. In a later work, anti-toxin release was combined with the production of an anti-biofilm factor (dispersin B) in the *P. aeruginosa*-infected murine model [90]. These systems reduced *P. aeruginosa* growth by 99% [89] and 98% [90], respectively.

#### Sensing of urinary tract infections (UTIs)

UTIs are among the most common bacterial infections. Clinical diagnosis of UTIs is challenging due to the limited reliability of the existing tests. Urine dipstick assay is a quick and inexpensive UTI test that screens for nitrite, leukocyte esterase, and blood [91]. Unfortunately, the sensitivity of this assay largely depends on the urine composition and can vary from 23.31%-72.28% [91]. Another detection method is based on detecting pyuria by urine microscopy. Pyuria is quantified by measuring the urinary leukocyte. However, leukocytes degrade quickly in urine that is not fresh or has not been appropriately preserved [92]. Another method directly detects pathogens by Gram staining followed by culture or other molecular tests to confirm a diagnosis. Detection by Gram staining under the microscope can be reliably positive if the concentration of bacteria in the urine is higher than 10^5^ CFU/mL, which is significantly above 10^2^–10^3^ CFU/mL, the threshold for early detection of UTIs [93]. Lastly, clinical diagnosis of UTIs by culture takes at least 2 days and is challenging to interpret [92, 94]. The interpretation of urine cultures that yield mixed flora in varying quantities can be challenging, as many potential combinations of microorganisms correlate with different types of UTIs.

BWCBs could overcome the sensitivity and response time limitations in the current techniques. For instance, rapid bioluminescence assays were developed and validated for detecting UTIs using *Photobacterium mandapamensis* or *Photobacterium leiognathi* bacteria [20]. Two platform technologies were developed: (i) tube bioluminescence extinction technology urine (TuBETUr) using *P. mandapamensis* and (ii) cellphone-based UTI bioluminescence extinction technology (CUBET) that uses *P. leiognathi* (Fig. 3D). The exposure of both platforms to infected urine resulted in rapid detection via loss of bioluminescence. Bioluminescence production requires oxygen. The pathogenic organisms present in urine consume oxygen, which will result in the inhibition of bioluminescence. TuBETUr detected UTI pathogens in less than 2 h for dilutions ranging from 10^2^–10^8^ CFU/mL of reference organisms in artificial urine and at less than 10^5^ CFU/mL pathogen concentrations in UTI-infected urine samples. CUBET could detect if a patient had a UTI within the first 5 min with high sensitivity, at less than 10^5^ CFU/mL, which is a lower cut-off limit for a positive UTI diagnosis. Moreover, it was shown that the *P. leiognathi* can be lyophilized and stored at room temperature for up to 3 months, indicating the potential for preserving the BWCB function until use in a point-of-care diagnostic setting. TuBETUr and CUBET are not strain or species-specific. Nevertheless, these assays were capable of providing information regarding pathogen presence and concentration and could help guide treatment decisions.

#### Biosensing of glucose level

Poorly managed blood glucose level adversely affects health. High blood glucose or hyperglycemia might be a symptom of diabetes mellitus. Whereas low blood sugar or hypoglycemia, might be fatal if left untreated. Available methods for monitoring blood glucose concentration can be divided into invasive (*i.e.*, blood sampling) and non-invasive (comprehensively reviewed in [95]). However, these methods have some limitations in accuracy, sensitivity, consistency, and stability [95, 96], and can be costly. Renal glycosuria is a rare condition wherein too much glucose is removed through the urine in the presence of normal plasma glucose levels. Urinalysis is the typical initial method of diagnosis. Techniques for measuring glucosuria are based upon either glucose oxidase or copper sulfate reduction and a variety of "stick" or "strip" tests. Most commercial semiquantitative urine tests can detect glucose in the urine only at a level of 50 to 250 mg/dl. Variations in the renal threshold among individuals can lead to significantly misleading data. Furthermore, the presence of different substances can alter the validity of tests for glycosuria [97].

A bacterial amperomteric biosensing system was developed for the detection of glucose [34]. *E. coli* was genetically engineered to display glucose dehydrogenase (GDH) with inaPb, the ice-nucleation protein (INP) from *Pseudomonas borealis,* as the anchoring motif. INP is used to display various proteins on the surface of Gram-negative bacteria without affecting cell viability [98]. The developed electrochemical microbial biosensor measures the current resulting from the oxidation of glucose and provides quantitative analytical information [99]. The amperometric biosensor allowed the determination of glucose with a detection limit (*i.e*., 0.4 µM) lower than other GDH-based glucose biosensors (*i.e*., 94 µM) [100], glucometer (*i.e.*, 0.56- 33 mM) [101] and other optical measurements for glucose monitoring [102]. In addition, excess saccharides (*e.g.,* L-arabinose and D-sucrose, D-xylose) and common interfering substances (*e.g.*, acetaminophen, ascorbic acid, and uric acid) did not affect the detection of glucose. Such BWCBs could be harnessed for the quantitative detection of glucose in a robust and cost-effective way.

In another work, digital amplifying switches and logic gates were utilized to enable transient recording and amplification of signals for glycosuria detection in clinical samples (Fig. 3E) [9]. Glucose is one of the primary carbon sources metabolized by bacteria; therefore, it is a challenging molecular signal to monitor using BWCBs. Courbet et al*.* selected pCpxP, a glucose, pyruvate, or acetate sensitive promoter, to derive the expression of red fluorescent protein (RFP) in *E. coli*. These BWCBs were encapsulated in polyvinyl acetate (PVA)/alginate hydrogel beads for containment and the beads were tested in urine samples of patients diagnosed with diabetes. The pCpxP switch beads reliably detected glycosuria in samples from diabetic patients, with a sensitivity of 88.9% and a specificity of 96.3%. This data demonstrates that digital amplifying genetic switches enable BWCBs to function in clinical assays and detect endogenous disease biomarkers [9].

### Biochemical assays

A biochemical assay is an analytical in vitro technique used to detect, quantify and/or study the binding or activity of the biomolecule of interest. Biochemical assays rely on biorecognition elements such as proteins, enzymes or antibodies that recognize the target analyte selectively. Although, both biochemical assays and biosensors recognize biomolecules, biosensors also have transducer elements, which process the biorecognition and convert it into a measurable signal. Bacteria-based biochemical assays combine low-cost production of a variety of biological elements for diagnosis, are fast, specific, and robust. Therefore, they fulfill the requirement of point-of-care testing.

Several types of biochemical assays have been described for the detection and diagnosis of diseases. Immunoassays are extensively used for in vitro diagnosis. This method measures a particular substance in a mixture via specific binding to an antibody. Antibodies can be produced for a wide range of analytes. Latex agglutination test (LAT) is a rapid immunoassay where the antibody is immobilized on latex particles to detect the presence of an analyte [103]. A biological equivalent of the latex agglutination test (LAT) was developed to detect extracellular analytes, providing the sensitivity and versatility of LAT and eliminating the need for expensive steps of antibody purification [19]. Combining bacterial surface display and antibody engineering, *E. coli* was engineered to display camelid nanobodies as a detection element (Fig. 3F). In this design, camelid antibodies were selected because they are heavy single-domain antibodies, are stably expressed in bacteria [104], and are functionally displayed on the outer membrane of *E. coli* [105]. As a proof of concept, a nanobody was first assessed against GFP and a tandem dimeric GFP analyte to induce cross-linking. This platform and a mathematical model of the agglutination process were used to determine the effects of nanobody expression level on the assay’s detection limit and the required number of bacterial particles in the assay. A whole-cell biochemical assay was developed to detect human fibrinogen (hFib), a clinically important biomarker. hFib is a glycoprotein that plays an important role in blood coagulation [106]. High levels of hFib are associated with cardiovascular disorder and inflammation [107, 108], while low levels indicate an increased risk of bleeding [106]. Two plasmids were constructed to display two different anti-hFib nanobodies (NbFib1 and NbFib2) in *E. coli*. First, the mathematical model predicted the range of analyte concentrations at which the agglutination zone would be observed. With this understanding, a diagnostic test was performed with purified hFib proteins in microplate agglutination assay format. The analyte-induced bacterial cross-linking, which was visible to the naked eye, enabled easy detection. Notably, the assay produced bacterial agglutination zones at the analyte concentration ranges consistent with the model prediction. In addition, a comparison of the assays using samples of hFib reconstituted in fibrinogen-deficient human plasma with samples reconstituted in phosphate-buffered saline (PBS) showed no significant difference between samples. These results suggest that the assay is robust to background variation. Moreover, this assay's detection limit was about 20,000-fold lower than that of the LAT assay for determining plasma fibrinogen [109]. Lastly, the detection limit of this assay was 30,000-fold and 15-fold lower than those of the piezoelectric agglutination sensor [110] and the amperometric immunosensor [111], respectively.

## Engineered bacteria as imaging vectors

Medical imaging plays a crucial role in the diagnosis, monitoring, and treatment of diseases. Imaging technologies enable noninvasive early detection of diseases, evaluation of disease progression, and treatment efficacy, all of which are crucial to favorable patient outcomes. Currently, several clinical imaging modalities exist, including nuclear medicine imaging, ultrasound, MRI, CT, optical imaging, and photoacoustic imaging. These techniques have been extensively reviewed elsewhere [112–114]. Enhanced sensitivity (*i.e.*, detection of positive imaging results when positive pathological results are present), specificity (*i.e.*, detection of negative results in patients free of the disease or injury), and spatial resolution of medical imaging modalities would enable earlier detection of diseases and improved monitoring of their progression or response to treatment. For instance, MRI has high spatial resolution and unlimited depth of penetration but has poor sensitivity and specificity. On the other hand, optical imaging techniques are highly sensitive but have a limited penetration depth, leading to low resolution [114]. Thus, there is a need to improve the visualization process with balanced characteristics of high sensitivity and specificity, high spatial resolution, and deep tissue penetration.

Recent advances in biotechnology have enabled the development of bacteria-augmented medical imaging. Bacteria can serve as contrast agents or imaging vectors to enhance the sensitivity and specificity of diagnostic imaging; however, the success of bacteria-augmented medical imaging hinges upon the localized accumulation of the bacteria at the disease site. Various bacterial species such as *E. coli* [115, 116], *Salmonella* [67, 117, 118], *Clostridium* [119, 120], and *Bifidobacterium* [121, 122] have been found to selectively colonize and replicate within solid tumors and in some case metastases. In addition, the probiotic strain *E. coli* Nissle 1917 has often been used for gastrointestinal (GI) applications due to its ability to colonize the GI tract [123–125]. Another important aspect is the disease-specific expression of contrast agents. The continuously improving understanding of the genetic mechanisms of many diseases has enabled the identification of key genes involved in or closely related to disease development. These genes are under the control of one or multiple promoters, and the activity of such promoter(s) might be solely specific to the pathologic process. Therefore, there is an exciting opportunity to use these promoters for bacterial expression of molecular probes/imaging reporters [126, 127] for disease-specific in vivo imaging. In this section, we describe the development of bacteria as imaging vectors for different imaging modalities, *i.e.*, nuclear medicine imaging (PET and SPECT), ultrasound, MRI, optical imaging, and photoacoustic imaging (Table 1). We also discuss the use of bacteria as tools for image-guided surgery.

**Table 1:** Engineered bacterial imaging vectors for various imaging modalities

Imaging Modality	Disease/Disorder	Bacteria	Imaging Agent	Reporter	Model	Refs
PET/SPECT	Melanoma	*S. Typhimurium*	[^124^I]FIAU		Mouse	[128]
	Mammary carcinoma	*E. coli* Nissle 1917	[^18^F]FEAU [^124^I]FIAU		Mouse	[129]
	Musculoskeletal bacterial infection	*Staphylococci sp Enterococci sp* *Proteus sp* *E. coli sp* *Pseudomonas sp*	[^124^I]FIAU		Human subjects	[130]
	Bacterial infection	*S. Typhimurium*	[^125^I]FIAU [^18^F]FLT		Mouse	[131]
	Bacterial infection	*E. coli* *S. aureus 29,213 and 25,923* *Streptococcus pneumoniae 49,619* *Enterococcus faecalis 49,532* *Staphylococcus epidermidis F362*	[^125^I]FIAU		Mouse	[132]
	Colon tumor	*C. novyi-NT* spores	[^125^I]FIAU		Mouse	
	Lung infection	*E. coli RS218*	[^125^I]FIAU		Mouse	[133]
Ultrasound	Gastrointestinal tract imaging	*E. coli* Nissle 1917	ARGs		Mouse	[134]
	Ovarian tumor	*S.* Typhimurium	ARGs		Mouse	
	Breast tumor	*E. coli* Nissle 1917	ARGs		Mouse	[135]
MRI	Kidney tumor	*M. magneticum* AMB-1	magnetite (Fe_3_O_4_) particles		Mouse	[136]
	Mammary tumor	*E. coli* Nissle 1917		Bacterioferritin	Mouse	[137]
	Pancreatic carcinoma	*C. novyi-NT*	iron-oxide nanoparticles		Mouse	[138]
	Liver carcinoma	*C. novyi-NT* spores	iron-oxide nanoparticles		Rat and rabbit	[139]
Fluorescence	DSS- induced Colitis	Gut commensal bacteria	D-amino acid hydroxycoumarin amino- D-alanine (HADA) 2-keto-3-deoxy-D-mannooctanoic acid (KDO) derivative 8-azido-8-deoxy-KDO (KDOAz) *N*-cyclopropenyl galactosaminyl carbamate (GalCCP)		Mouse	[140]
	Mammary tumor	*E. coli*	CytoCy5S		Mouse	[141]
	Colon tumor	*B. Breve*				
	Bacterial infection	*S.* Typhimurium				
	Bacterial infection	*S. aureus*	UBI29-41-IG02		Mouse	[142]
	Bacterial infection	*E. coli*	Maltodextrin-based imaging probes		Rat	[143]
	Prostate tumor	*S.* Typhimurium A1		RFP (in PC-3 cell) GFP	Mouse	[144]
	Fever	*E. coli*		GFP (mWasabi)	Mouse	[145]
Bioluminescence	Intracolonic adenocarcinoma	*E. coli*		Luciferase (Lux)	Mouse	[146]
	Colon carcinoma Lung metastases	*S.* Typhimurium ΔppGpp		*Renilla luciferase variant 8*	Mouse	[147]
	Pancreatic ductal adenocarcinoma	*S.* Typhimurium VNP2009		mKate2 (in CFPAC-1 cell) luciferase	Mouse	[148]
	Infection model	*S. aureus*		Modified *lux* operon	Mouse	[149]
	Thigh model of infection	*E. coli*		*Luciferase*	Mouse	[150]
	Catheter-based infection	*S. aureus* *P. aeruginosa*		*Luciferase*	Mouse	[151]
	Bacterial implant infection model	*S. aureus Xen29*		*Luciferase*	Mouse	[152]
	Infarcted myocardium	*S.* Typhimurium ΔppGpp		*Renilla* luciferase gene *(RLuc8)*	Mouse	[153]
Photoacoustic imaging	Colon tumor Breast tumor	*E. coli* BL21		Melanin-pDA-ICG	Mouse	[154]
	Breast tumor	*E. coli*		Phytochrome-based reporter protein (m*Dr*BphP-PCMm/F469W)	Mouse	[155]
	Melanoma	*L. lactis*		GFP mCherry IRFP713 β-galactosidase	Mouse	[156]
	Breast tumor Gastrointestinal tract	*E. coli* *S. aureus* *M. luteus* *P. aeruginosa*	Modified gold nanoparticles with glucose polymer		Mouse	[157]

### Nuclear medicine imaging

Positron emission tomography (PET) and single photon emission computed tomography (SPECT) are the two types of nuclear medicine imaging. PET and SPECT are highly sensitive with limitless penetration depths. However, they have low spatial resolutions (5–7 mm for clinical PET and 8–10 mm for clinical SPECT) [114]. They quantitatively measure metabolic and biochemical processes [158] and are used in diagnosing and managing cancer, cardiovascular disease, and brain disorders [159, 160]. PET and SPECT are based on detecting the radioactivity of an intravenously administered radioactive tracer [160]; thus, localization of the radiotracers at the disease site is of utmost importance. Radiotracers accumulate in tumors (*e.g.*, ^18^F-FDG [161], ^18^F-FDM [162]) or inflamed regions (*e.g.*, ^68^ Ga-DOTATOC [163]) or can bind to specific proteins (*e.g.*, ^11^C-PK11195 [164]). Unfortunately, certain radiotracers, such as ^18^F-FDG, have limited specificity and accumulate at infectious and aseptic inflammation sites, which may result in inaccurate diagnosis [165].

To enhance specificity for cancer imaging, PET/SPECT was combined with genetically encoded bacterial imaging probes for tumor visualization. For instance, the simplex virus type 1 thymidine kinase (HSV1-TK) genetic sequence was designed to be highly selective to radiolabeled nucleoside analogs. The HSV1-TK encodes for an enzyme that specifically phosphorylates the radiolabeled tracers, resulting in their entrapment and accumulation within host cells [128] and facilitating highly specific tumor imaging. Several tumor-targeting bacteria were successfully PET scanned using this strategy. Attenuated *S.* Typhimurium expressing HSV1-TK was imaged with ^124^I-2’-fluoro-1-β-D-arabino-furanosyl-5-iodo-uracil ([^124^I]FIAU) to detect melanoma in mice [128]. *E. coli* Nissle 1917 with endogenous thymidine kinase (TK) was successfully imaged with ^18^F-2′-Fluoro-2′deoxy-1-β-D-arabinofuranosyl-5-ethyl-uracil ([^18^F]FEAU) and [^124^I]FIAU for the detection of breast tumors [129]. These studies demonstrated that the magnitude of radioactivity (image intensity) correlates with the number of bacteria that colonizes the tumor. Engineering bacteria to be trackable using PET/SPECT also provides an opportunity for PK/PD evaluation of bacteria-based cancer therapy. Currently, tissue sample collection is the most commonly used technique for quantitative evaluation of bacterial accumulation in the target tissue. This imaging technique would be of considerable value in clinical applications to identify sites of solid tumors, noninvasively monitor the targeting and proliferation of bacteria, and evaluate bacteria-based therapy.

**Fig. 4:**
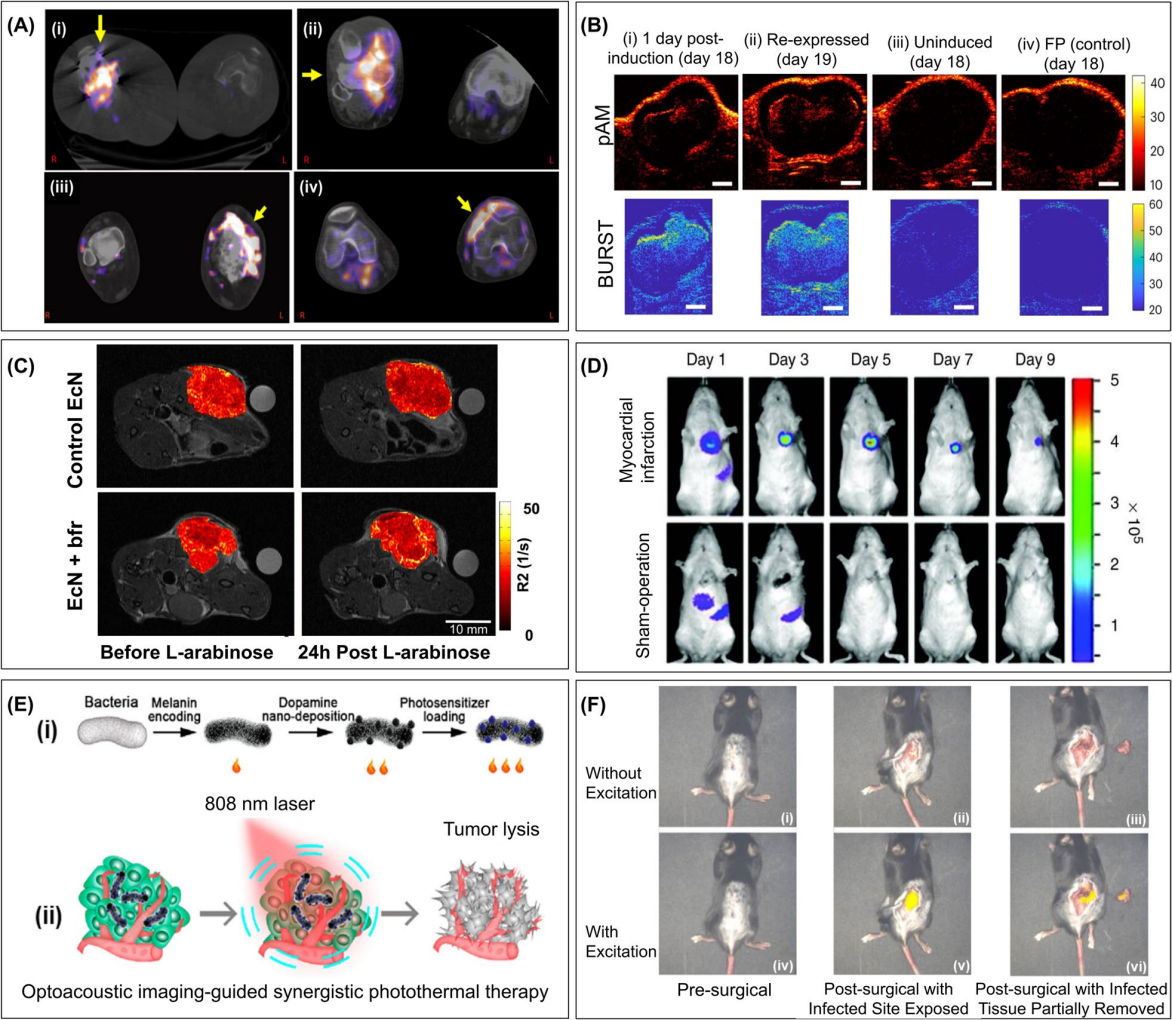
Bacteria as imaging vectors. **A** Super-imposed PET and CT images of established infection 2 h post [^124^I]FIAU radiotracer administration in human patients. (i) methicillin-resistant *Staphylococcus aureus* (MRSA)-caused septic arthritis (right knee with a prosthetic joint), (ii) MRSA-induced septic arthritis (right knee with history of chronic MRSA osteomyelitis), (iii) cellulitis (left lower extremity with history of osteomyelitis in the left distal tibia), (iv) necrotizing MRSA osteomyelitis of the proximal tibia (left knee). Reprinted with permission from [130]. **B** In situ bARG_Ser_ expression by *E. coli* Nissle 1917 (EcN) for ultrasound imaging of tumor colonization. Representative parabolic ultrasound (pAM) and Burst Ultrasound Reconstructed with Signal Templates (BURST) images of tumors colonized by bARG_Ser_-expressing EcN (i) at 1 day after induction with intraperitoneally injected L-arabinose (on day 18 post subcutaneous injection of tumor cells and day 4 post tail vein injection of EcN bacteria), (ii) at 1 day after the collapse of gas vesicles and re-injection of L-arabinose for re-induction (day 19), (iii) uninduced on day 18, (iv) pAM and BURST images of tumors colonized by fluorescence protein-expressing (FP) EcN after L-arabinose induction. Reprinted with permission from [135]. **C** *R2* relaxation maps of mammary tumors obtained before and after L-arabinose induction of bacterioferritin (bfr) expression. Tumor-bearing mice were injected with control bacteria (upper panel) or P_BAD_-*bfr* encoding EcN (lower panel). Left and right panels are before and 24 h after injection of L-arabinose, respectively.. Reprinted with permission from [137]. **D** In vivo bioluminescence imaging of *S.* Typhimurium ΔppGpp tropism for myocardial infarction. Time course study of bioluminescence after intravenous injection of bacteria in mice with or without myocardial infarction. Reprinted with permission [153]. **E** Photoacoustic imaging-guided synergistic photothermal therapy. (i) Illustration of the preparation of ternary photosensitive bacteria by combining genetic engineering for melanin expression with surface co-deposition of polydopamine and synthetic indocyanine green (ICG). (ii) Monochromatic irradiation at 808 nm results in stable triple photoacoustic and photothermal effects for imaging and therapy. Reprinted with permission from [154]. **F** Acute spinal implant infection excision under the fluorescence image-guided surgery system. Analysis of images without (i-iii) and with (iv-vi) fluorescence excitation at 680 shows no fluorescence signal prior to skin incision (i) and (iv); the presence of strong fluorescence signal with excitation post skin incision (v), and reminiscent fluorescence signal post partial excision of infected tissue (vi). Reproduced with permission [167]

The reporter probe [^124^I]FIAU has also been used for the imaging of musculoskeletal bacterial infections in human subjects (Fig. 4A) [130]. Gram-positive (*Staphylococci sp., Enterococci sp.*) and gram-negative (*Proteus sp., E. coli sp., Pseudomonas sp.*) organisms were identified. In addition, (^125^I)-FIAU and 3′-deoxy-3′-[^18^F]fluorothymidine ([^18^F]FLT) have been assessed for noninvasive PET imaging of bacterial infections [131] by targeting the endogenous TK with radiotracers. Furthermore, [^[25^I]FIAU allowed in vivo imaging of intratumoral infections and localized bacterial infection of muscle [132]. [^125^I]FIAU was also used to image and quantify bacterial load in a preclinical lung infection model with *E. coli RS218* [133]. In vitro*,* experiments revealed significant binding of [^125^I]FIAU to wild-type *E. coli* (80%) and only 9% binding to TK-negative bacteria [166]. Indeed, targeting the endogenous TK with radiolabeled nucleoside analogs provides a unique approach to the noninvasive diagnosis of bacterial infections as TK is common to multiple pathogenic bacterial strains. Although this approach is not selective to a specific pathogen, it enables distinction of sterile inflammation from bacterial infection. This technique can be extended to detect infections affecting other anatomical sites and microorganisms possessing a suitable thymidine kinase.

### Ultrasound imaging

Ultrasound imaging uses high-frequency sound waves to view different biological tissues. Ultrasound is a widely available and affordable technique with deep tissue penetration (mm to cm) and high spatial (0.01–2 mm) and temporal (seconds to minutes) resolution [103]. The choice of frequency is important since higher frequencies give increased spatial resolution but reduced penetration depth [168]. Ultrasound images can be acquired in “real-time,” thus, providing immediate visual guidance. However, ultrasound is limited by the difficulty in imaging bones and air-filled structures like lungs and the depth of penetration compared to imaging techniques with unlimited depth (*e.g.*, PET and SPECT). Currently, microbubble contrast agents (MCAs) are being used to improve ultrasound sensitivity, but for targeted imaging applications, limited localization of MCAs limits imaging sensitivity [169, 170].

Engineered bacteria were developed to enhance contrast in ultrasound imaging. Gas vesicles (*i.e.*, gas-filled protein nanostructures) were genetically encoded from acoustic reporter genes (ARGs) [134] in bacteria. These gas vesicles are stable and have a long half-life. Due to their air-filled interiors' low density and high compressibility compared to surrounding tissues, gas vesicles can scatter sound waves, producing robust ultrasound contrast. This novel approach was first implemented for imaging the gastrointestinal tract, which is typically difficult to visualize due to its location deep inside the body. For this purpose, the probiotic strain *E. coli* Nissle 1917 was transformed with ARG-expressing plasmid and introduced into the colons (center or periphery of the colonic lumen) of anesthetized mice. Ultrasound images clearly revealed the localization of ARG-expressing *E. coli* cells in the administered region. The same genetic construct was adapted for expression in *S.* Typhimurium and showed that these cells could be imaged after intratumoral injection into a mouse ovarian tumor [134]. In a separate study, two ARGs with ninefold and 38-fold stronger non-linear contrast than their predecessors were developed and implemented in bacteria and mammalian cells, respectively [135]. Bacterial ARG was expressed in the *E. coli* Nissle 1917 and enabled ultrasound imaging (6 mm focus, frequency of 15.625 MHz) of these bacteria colonizing an orthotopic breast tumor (Fig. 4B). To address limited sensitivity in the visualization of ARG-expressing cells, Burst Ultrasound Reconstructed with Signal Templates (BURST), an ultrasensitive imaging paradigm tailored to ARGs, was developed [171]. BURST improves the detection limit by over 1,000-fold over the previously reported concentrations of 5 × 10^7^ cells ml^−1^ with ARG-expressing cells [134]. This lower detection limit is particularly beneficial in probiotic therapy, where bacteria are administered at very low concentrations and cannot be imaged until several days after replication in the target tissues.

### Magnetic resonance imaging (MRI)

MRI is an imaging method with a high spatial resolution (25–100 μm (preclinical) and ∼1 mm (clinical)), a temporal resolution of minutes to hours, and unlimited tissue penetration depth [114]. MRI is a routine diagnostic tool for cancer, brain injury, spinal cord disorders, stroke, and musculoskeletal injuries. To enhance in vivo imaging contrast, contrast agents (comprehensively reviewed in [172]) are administered to patients. Organ-specific contrast agents (*e.g.*, liver, spleen, brain lesion, and lymph nodes) are available for commercial use, and more are being evaluated in human clinical trials (reviewed in [172]). Selective uptake and accumulation of contrast agents in the target tissue are critical to the sensitive depiction of pathology. The innate tumor-homing attribute of bacteria has been harnessed to accumulate magnetotactic bacteria-based MRI contrast agents to cancerous tissues selectively. These bacteria naturally produce small magnetite particles (magnetosomes), which can be utilized as MRI contrast agents [173]. *Magnetospirillum magneticum* AMB-1 was found to selectively colonize murine kidney tumors and enhance positive MRI contrast of tumors by up to 1.39 folds [136]. These results were confirmed with PET using ^64^Cu-labeled AMB-1. Thus, these bacteria could improve MRI sensitivity in cancer diagnosis and treatment evaluation preclinically. Another strain of magnetotactic bacteria, *Magnetococcus marinus* MC-1, was guided by an external magnetic source to penetrate tumors and transport drugs into hypoxic regions of the tumor [174].

The bacterial iron storage proteins can also be used as MRI reporter genes. Bacterial ferritin-like proteins were overexpressed in probiotic *E. coli* Nissle 1917. Tumor-specific induction of bacterioferritin expression in colonized mammary tumors resulted in significant contrast changes that can be monitored by MRI [137] (Fig. 4C). In another approach, labeling the surface of non-magnetotactic bacteria with MRI contrast agent nanoparticles enabled their visualization via MRI. For example, *Clostridium novyi-*NT bacteria were labeled with iron-oxide nanoparticles and were percutaneously injected in a pancreatic carcinoma mouse model [138]. This study demonstrated the potential of MRI in visualizing intratumoral delivery and distribution of these iron-oxide nanoparticle-labeled *C. novyi-*NT. Similarly, in another study, iron-oxide nanoparticle-labeled *C. novyi-NT* spores were successfully used for MRI-monitored locoregional delivery to liver tumors in rabbit and rat orthotopic models and a comparison of biodistribution resulting from intratumoral versus intra-arterial injections [139].

### Optical imaging

#### Fluorescence imaging

Fluorescence imaging (FI) is an emerging imaging technique for clinical care [62, 175]. This method is highly sensitive but has a limited depth of penetration (∼1 cm) and low spatial resolution (2 -3 mm) [62]. FI has been demonstrated for the assessment of retinal vasculature, lymph node mapping, and tissue perfusion [176–178]. The first Food and Drug Administration (FDA) breakthrough therapy designation was given to pegloprastide (AVB-620) for intraoperative detection and visualization of positive margins during breast cancer surgery [179]. Current efforts are primarily focused on improving the specificity of imaging contrast agents for the visualization of anatomic structures (e.g., nerves), assessment of treatment response [180], delineation of tumors for image-guided surgery (discussed in Sect. 2.6) [181], and detection of malignant lesions and regional metastases [182].

Bacteria selective accumulation at the disease sites (discussed in Conclusions and outlook section) has been combined with genetic engineering to incorporate a fluorescent protein reporter gene to provide a targeted and noninvasive tool for detection and monitoring. Bacterial expression of near-infrared fluorescent probes enables the detection of fluorescence signals from deeper tissues and offers the possibility for clinical translation [183].

Due to the intrinsic cancer-targeting ability of bacteria, many research works have focused on leveraging FI for bacteria-based cancer detection and therapeutic efficacy monitoring. This approach enabled the detection of tumor growth and metastasis, thus, minimizing the need for repetitive biopsy. For instance, *S.* Typhimurium A1, auxotrophic for leu-arg, was isolated with increased targeting ability for tumor cells [144]. RFP-expressing prostate cancer (PC-3) cells were injected subcutaneously into the flank of the nude mice. GFP-expressing *S.* Typhimurium A1 bacteria were administered either intravenously or intratumorally. Whole-body fluorescence imaging allowed the observation of tumor growth via RFP labeling and bacteria distribution via GFP labeling. Results showed selective colonization of *S.* Typhimurium in PC-3 tumors and complete clearance from normal tissues. In addition, treatment with *S.* Typhimurium A1 inhibited tumor growth and led to complete tumor regression within 15–26 days after starting the treatment.

Engineered bacteria for FI have been utilized in other applications as well. An elevated body temperature indicates a bacterial or viral infection and certain inflammatory conditions. In a recent study, *E. coli* was engineered to sense physiologically relevant temperature ranges [145]. Two thermal bioswitches were developed using the operator-promotor TlpA, a temperature-dependent protein from the virulence plasmid of *S.* Typhimurium, and TcI, a temperature-sensitive variant of the bacteriophage λ repressor cI. GFP (mWasabi) was expressed in Mach1 *E. coli* under the control of these repressors. Both TlpA and TcI bioswitches responded to temperature with hundreds-fold changes in gene expression. The operator-promotor TlpA was then selected to be characterized. *E. coli* expressing TlpA_36_, a high-performance bioswitch centered at 36 °C, was subcutaneously injected into both hindlimbs of a nude mouse. Next, the temperature was locally elevated using focused ultrasound treatments. In response to increased temperature, the engineered bacteria produced a GFP signal detected by in vivo fluorescence imaging. The same temperature sensor was also used to demonstrate localized fluorescence expression in mice in response to a housing temperature increase. Finally, the genetically engineered *E. coli* was contained in vivo through the acceleration of cell death at nonpermissive temperatures (*i.e.*, 25 °C). This allows self-destruction after fecal elimination and prevents environmental escape.

In other approaches, bacterial proteins and mechanisms were harnessed for FI without resorting to engineering. The available fluorescent probes include (i) bacterial surface-targeted fluorophores that bind to the surface of bacteria [140], (ii) enzyme-activated probes that are reduced to a fluorescent form by bacterial-specific endogenous nitroreductase enzyme [141], (iii) antimicrobial peptides which can bind to or are taken up by specific bacteria [142], and (iv) metabolism-based probes where fluorophore-labeled metabolic substrates may be specifically incorporated by bacteria [143].

An example of surface-targeted fluorophores used the metabolic D-amino acid-based labeling method to tag bacterial peptidoglycan (PGN) fluorescently [140]. Mutation in PGN receptors has been correlated with IBDs [184]. The fluorescent D-amino acid hydroxycoumarin amino-D-alanine (HADA) was administered to mice, where they will be covalently attached to the PGN stem peptide during bacterial cell wall construction. Commensal bacteria in the small intestine were quickly labeled (within 45 min) but washed out within 3 h, whereas maximal labeling was within 3 to 8 h for the caecum and colon. Multiphoton intravital microscopy allowed real-time visualization of the dynamics of the host-microbiota interaction. Moreover, it showed bacterial movement within the lumen and adhesion to the mucosal surface of the epithelial barrier. In addition, more immunomodulatory surface molecules, *i.e.*, lipopolysaccharide (LPS) and capsular polysaccharide (CPS), were labeled using 2-keto-3-deoxy-D-mannooctanoic acid (KDO) derivative, 8-azido-8-deoxy-KDO (KDOAz) and *N*-cyclopropenyl galactosaminyl carbamate (GalCCP), respectively. All three aforementioned immunomodulatory surface components (i.e., PNG, LPS and CPS) were labeled on exogenous bacteria and simultaneously introduced and imaged within the intestinal lumen of the murine host. Multiphoton intravital microscopy enabled tracking of all three surface components as the bacteria crossed the lumen and interacted with the host epithelial cells. This method is valuable for understanding and monitoring intestinal diseases [140].

Endogenous bacterial enzymatic activity can be harnessed to activate an exogenously administrated fluorescence imaging probe. Nitroreductase (NTR) enzymes were exploited to reduce the red-shifted, quenched fluorophore CytoCy5S to a fluorescent form for imaging [141]. NTR enzymes are a family of bacterial enzymes capable of reducing nitro functional groups. *E. coli*, constitutively expressing luciferase, was injected intravenously into mice bearing subcutaneous mammary xenograft tumors, and CytoCy5S was intraperitoneally administered. Overlay of luminescence and fluorescence confirmed that the activation of CytoCy5S occurred specifically at the tumor site. Activation of CytoCy5S was also demonstrated in the Gram-positive *Bifidobacterium Breve* intratumorally injected into mice bearing subcutaneous colon flank tumors. Three days post-injection of *B. Breve*, mice received an intraperitoneal injection of CytoCy5S. The fluorescent signal was confined to the tumor location. In addition, in vivo tracking of *S*. Typhimurium UK-1 infection was performed. Mice received an intravenous injection of luminescent *S*. Typhimurium. Progression of infection was monitored by bioluminescence to a chronic phase, and then mice received an intraperitoneal injection of CytoCy5S. Fluorescence imaging showed a strong signal from the liver and spleen. Comparison with an NTR-knockout confirmed the enzyme specificity to the probe. This system allowed the noninvasive detection of Gram-negative and Gram-positive bacterial localization in vivo*.*

An antimicrobial peptide fragment UBI29-41-based near-infrared fluorescent imaging probe was developed for imaging *Staphylococcus aureus* infection in a murine local infection model [142]. The probe is composed of UBI29-41 conjugated to a near-infrared dye ICG-Der-02 (UBI29-41-ICG02), where UBI29-41 is a cationic human antimicrobial peptide that targets the anionic surfaces of bacterial cells without leukocytes’ activation, and ICG-Der-02 is a hydrophilic ICG derivative dye with deep tissue penetration and fast clearance rate. To establish the infection model, *S. aureus* was subcutaneously injected into the axillary fossa and lower flank of mice. UBI29-41-IG02 was injected into the tail vein when significant swelling was visible at the infection site (*i.e*., 24 h after *S. aureus* inoculation). In vivo NIR imaging showed the fluorescence signals of the probe initially spread all over the body and then cleared by the renal pathway after 6 h. The probe accumulated selectively at the infection site and allowed identification of the infection within 1-h post-injection of the probes. Previous studies showed that UBI29-41-based imaging might be promising in distinguishing between sterile and infectious inflammations [185, 186]. Therefore, the UBI29-41-ICG02 imaging probe may be a promising imaging agent for detecting bacterial infections.

Bacteria metabolism can be harnessed for the uptake of contrast agents and in vivo imaging. For instance, the bacteria-specific maltodextrin transporter pathway can internalize maltodextrin-based imaging probes (MDPs). MDPs are composed of a fluorescent dye (*i.e*., perylene for MDP1 or IR786 for MDP2) conjugated to maltohexaose, a major source of glucose for bacteria. MDPs are highly specific to bacteria since mammalian cells do not express the maltodextrin transporter and cannot uptake contrast agents conjugated with maltohexaose. The efficacy of MDP2 for imaging bacterial infections in rats was assessed [143]. The rats were injected in the left and right thigh muscles with *E. coli* and saline, metabolically inactive *E. coli*, or lipopolysaccharides (as control). MDP2 was intravenously injected after 1 h. Fluorescence imaging showed specific probe accumulation at the infection site and that MDPs enable visualization of as few as 10^5^ CFU. In addition, MDPs can distinguish between active bacteria and inflammation induced by either metabolically inactive bacteria or lipopolysaccharides. In this study, MDPs enabled in vivo imaging of bacterial infection with a unique combination of high sensitivity and specificity for bacteria without the need for delivering large quantities of imaging probes.

#### Bioluminescence

Bioluminescence imaging involves the genetic engineering of bacteria to incorporate a bioluminescence reporter gene such as luciferase to enable bacteria visualization. Bioluminescence is highly sensitive; however, it has a low resolution (3- 5 mm) and a limited depth of penetration (1- 2 cm) [114]. In contrast to fluorescence, bioluminescence does not require excitation light. As a result, there are no phototoxicity or background noise (auto-fluorescence) issues. In fact, bioluminescence imaging can achieve a very low background due to the lack of natural bioluminescence from tissues; thus, it has a superior signal-to-noise ratio (higher sensitivity) over fluorescence imaging [114].

Bioluminescent bacteria have been developed to target various diseases, enabling noninvasive visualization and monitoring. Leveraging the intrinsic tumor-selective colonization in *E. coli* [129, 146, 187, 188] and *S.* Typhimurium [117, 147], these microorganisms have been engineered for the expression of bacterial luciferase (*Lux*). The bioluminescence signal permitted the localization of the bacteria in tumors and metastases and allowed stable imaging in mouse models over several days. In orthotopic murine intracolonic adenocarcinoma models, it was determined that *E. coli* is traceable as long as the population size is larger than 10^4^ CFU/g. Bioluminescence signals in liver metastases also enabled the visualization of metastatic nodules. Quantification of *E. coli* in various organs and grafted tumors showed a bacterial load of ~ 10^8^ CFU/g in the primary tumor 4 days post-injection, which was maintained for 8 days. Bacterial loads in the liver, spleen, and lung decreased and were undetectable after 4 days post-injection [146].

*S.* Typhimurium ΔppGpp is defective in the synthesis of 5′-diphosphate-3′-diphosphate (ppGpp) due to the deletion of *relA* and *spot* genes [189], which significantly increases the half-lethal dose of ppGpp in mice by 100,000—1,000,000-fold [190]. In addition, the functional loss of *Salmonella* virulence genes [189] allows *S.* Typhimurium ΔppGpp to serve as an ideal vector for targeted delivery [191, 192]. *S.* Typhimurium ΔppGpp was engineered to carry a dual inducible gene expression cassette encoding an antitumor protein (cytolysin A) and a bioluminescence reporter gene (*renilla* *luciferase* (*rluc8*)) [147]. A 3 × 10^7^ CFU aliquot of the engineered *S.* Typhimurium ΔppGpp was intravenously injected into the murine colon carcinoma model. Bacterial bioluminescence was detected only in the tumor tissue upon administration of the inducer, doxycycline (Doxy), at 3 days post-injection. In addition, results showed significant suppression of tumor growth. To evaluate the localization to lung metastases, CT26 cells, the colorectal carcinoma cell line, were injected at 5 × 10^4^ into mice via the tail vein. Successful gene expression of the engineered *S.* Typhimurium in targeted lung metastases was confirmed by monitoring RLuc8 activity after Doxy induction. Mice treated with engineered bacteria in the presence of Doxy showed a 37% and 23% reduction in the growth of metastases compared to the controls mice treated with PBS or without Doxy, respectively.

A fluorescence-luminescence tracking system was developed to monitor the bacteria-based suppression of pancreatic ductal adenocarcinoma (PDAC), a highly aggressive lethal malignancy with a poor prognosis [148]. The human pancreatic cancer cells CFPAC-1 were engineered to stably express the far-red fluorescent protein, mKate2, by lentivirus infection. *S.* Typhimurium strain VNP20009 was engineered for the expression of luciferase. CFPAC-1 was injected subcutaneously into the right flank of nude mice. When the tumor size reached 100 mm^3^, *S.* Typhimurium was intratumorally injected. Fluorescence and bioluminescence imaging enabled concomitant tracking of xenograft pancreatic tumor growth, metastasis status, and drug distribution in live mice. Furthermore, *S.* Typhimurium VNP20009 promoted tumor apoptosis in vivo. The far-red fluorescent signal from the cancer cells in the treated group was lower than that of the control group (PBS-treated), contrary to the intense bacterial bioluminescence signal from colonized tumors. This dual imaging system offers a valuable tool for noninvasive live imaging of solid tumors and monitoring of bacteria-based treatment.

Another application of bioluminescence is the imaging and detection of infarcted myocardium. *S.* Typhimurium ΔppGpp was reported as an imaging vector for myocardial infarction in rats [153]. This technology demonstrated for the first time the ability of this bacterial strain to colonize infarcted myocardial tissue (Fig. 4D). No sign of severe local or systemic immune reactions was noted following i.v. administration of *S. Typhimurium* ΔppGpp. The examination of the infarct size before and after infection did not show a significant difference. The mechanism of bacterial targeting of infarcted myocardium is not elucidated yet. It is suggested that the inflammatory environment could attract *S.* Typhimurium ΔppGpp or that the infarct provides a favorable environment for proliferation. Infarcts are routinely detected by nuclear imaging techniques; however, the relatively low resolution of the images limits the detection of small areas of infarction [193]. Bioluminescent bacteria enabled real-time imaging of the infarcted myocardium without leakage to noncardiac tissue. *S.* Typhimurium ΔppGpp had no adverse effects. Furthermore, antibiotic treatment resulted in a complete clearance of *Salmonella* within 2 days.

Beyond disease detection, bioluminescence-based imaging technology can contribute to a better understanding of disease development and drug screening. For example, bioluminescent bacteria are developed for real-time noninvasive monitoring of implant infection models to elucidate the biofilm development process. Biomaterial-associated infections are a major cause of implant failure. These infections are caused by microbial biofilm formation on implant surfaces with increased resistance to antibiotic therapy and immune response [194]. Therefore, treatments are often inadequate to resolve the problem, necessitating surgical removal and replacement of the implant. The development of bioluminescent biofilm-forming pathogens enabled the validation of infection models in vivo [149, 150] and on catheter lumen in a murine model [151]. A bioluminescent bacterial implant infection model permitted obtaining quantitative information on bacterial spread into the surrounding tissues and enabled monitoring of the infection process, including interactions with the host defense system [152]. Combining bioluminescence-based technology with implant infection models provided a more accurate assessment of the pathogenicity of implant-related infections. This model is well suited for rapid screening of novel antimicrobial compounds for implant infections.

### Photoacoustic imaging

Photoacoustic imaging is an emerging optical imaging technique that combines optical excitation and acoustics detection to overcome the limitations of deep-tissue imaging. One of the main advantages of photoacoustic imaging is its high resolution (0.01–1 mm). Although photoacoustic imaging has a better penetration depth (∼ 7 cm) than most optical imaging, its penetration depth is still restricted compared to MRI and nuclear imaging techniques [114]. Photoacoustic imaging provides multiscale imaging capacity, bridging the gap between the microscopic and macroscopic realms with the same type of contrast. Other unique advantages of this imaging technique are label-free imaging of different tissue types, compatibility with a wide variety of contrast agents including near infrared dyes (*e.g.,* IRDye800CW, ICG, AF750), bioinorganic nanoparticles (*e.g.,* Au or CNT), and fluorescent proteins (*e.g.,* iRFP), and the ability to identify chemical composition of biological samples [195]. Clinical applications of photoacoustic imaging include breast [196] and skin [197] cancer imaging, musculoskeletal imaging and inflammatory arthritis detection [198] and gastrointestinal tract imaging [199]. Genetically encoded photoacoustic probes can offer the advantages of better penetration and high spatial resolution to precisely monitor pathological and physiological changes in vivo. Recently, the integration of a monochromatic irradiation-mediated ternary photoacoustic and photothermal system was reported in *E. coli* BL21 [154]. *E. coli* was genetically engineered to express melanin and then co-deposited with dual synthetic indocyanine green (ICG), a medical dye, and polydopamine nanoparticles (pDA) via in situ polymerization on the bacterial surface (Fig. 4E). All components of the formed ternary combination share an excitation wavelength of 808 nm, which generates a stable triple photoacoustic and photothermal effect under monochromatic irradiation. The engineered bacteria were administered into colon and breast subcutaneous tumors in mice and enabled the evaluation of treatment effectiveness. In addition, the ternary combination BL21 significantly improved tumor regression and animal survival. Furthermore, to improve the detection sensitivity and remove the background signal, a genetic reporter protein (m*Dr*BphP-PCMm/F469W) with high photoswitching properties was engineered in *E. coli* [155]. Photoswitching contrast is defined as the ratio of the absorption coefficient at the ON state to that of the OFF state. Therefore, a high photoswitching contrast indicates efficient background suppression and better image specificity. A custom-built photoacoustic-computed tomography system was employed, achieving a resolution of 213-μm in lateral and 150-μm in axial directions in vitro*. *Ex vivo analysis was performed on chicken breast to mimic the deep biological tissue environment. Photoswitching feature of *E. coli* was examined at different depths and results revealed good photoswitching efficiency between the two states in deep tissue up to a centimeter scale. This method allowed imaging in murine breast tumor model over seven days and permitted photoacoustic monitoring of deep-tissue tumors via reduction of background signals [155].

Melanoma is the most severe type of skin cancer with a poor prognosis and high mortality rate. Current therapeutic approaches are unable to target tumor cells due to increased stiffness resulting from abnormal extracellular matrix structural changes and a hypoxic tumor environment [200]. In this regard, the ability of *L. lactis* to detect melanoma using Multispectral Optoacoustic Tomography (MSOT) was investigated [156]. *L. lactis* was engineered to express β-galactosidase, a well-characterized MSOT contrast agent [201], under the Stress-Inducible Controlled Expression (SICE) system. This system ensures the expression of the gene of interest is induced under stress conditions and does not need exogenous induction nor the presence of a regulatory gene [202]. Bacteria-enabled detection of melanoma in vivo was evaluated using *L. lactis*-β-gal *and L. Lactis*-IRFP713 and athymic mice implanted with melanoma cells subcutaneously. *L. lactis*-β-gal *and L. lactis*-IRFP713 were injected intratumorally for MSOT and near-infrared (NIR) imaging of mice bearing skin tumors, respectively. The reconstructed MSOT images (center frequency 15 MHz) and NIR imaging data revealed that *L. lactis* localize preferentially within the tumor hypoxia niches. These results suggest that *L. lactis* is a promising tumor-targeting agent to deliver therapeutic molecules into the tumor hypoxic microenvironment without the need of induction.

Exogenous probes are also used to improve photoacoustic imaging sensitivity and signal strength in *E. coli* (ATCC 11303), *S. aureus* (patient isolate), *Micrococcus luteus* (BNCC 102589) and *P. aeruginosa* (BNCC 125486). Glucose polymer (GP) was conjugated to the surface of gold nanoparticles and modified with diazirines molecules and chlorin e6 (Ce6) for enhanced aggregation. The 11 nm gold nanoparticles were demonstrated to aggregate into ~ 150 nm clusters upon laser irradiation (405 nm, 1.0 W cm^−2^, 25 min) ex vivo. The modified gold nanoparticles were taken up by bacteria as a carbon source. The developed strategy was evaluated in proof-of-concept models of breast cancer tumor xenografts and gastrointestinal tracts [157]. For the breast cancer model, bacteria were subcutaneously injected into the left and/or right thigh region of mice. For the gastrointestinal tract model, agarose gel containing *E. coli* was injected into the gut lumen. Next, the gold nanoparticles were intravenously injected and subsequently taken up by bacteria as the carbon source through a bacteria-specific ABC transporter pathway. Aggregation of the GP-conjugated gold nanoparticles with the bacterial cells resulted in enhanced photoacoustic signals (~ 15.2-fold increase) and enabled sensitive imaging of bacteria in vivo. This technique allowed the localization of as few as 10^5^ CFU/mL of bacteria, around two orders of magnitude lower than most optical contrast agents.

### Image-guided surgery

Surgeons usually rely on their experience, touch, and sight to make critical decisions during surgical procedures. The advent of Image-Guided Surgery (IGS) has allowed surgeons to make more informed decisions, operate with more precision, and improve outcomes. Most IGS approaches use cell-specific tracers in conjunction with dedicated imaging systems. In oncological procedures, these targeted tracers bind to their tumor-associated targets after passive tissue diffusion [203, 204]. However, the diffusion of the tracer through the target tissue is limited due to the physiological traits and characteristics of the tumor microenvironment [205]. Immunohistochemical evaluation of resected tumors has shown that the tracer distribution throughout the tumor can be heterogeneous and superficial [205]. An alternative approach would be to use tracers that can actively target and penetrate cancerous tissue. In this regard, bacteria present distinctive advantages of selective colonization and replication in cancerous, inflamed, or infarcted lesions.

A spine implant infection model of the bioluminescent *S. aureus* Xen36 was studied using an ^89^Zr-NIR680-1D9 dual-labeled probe, a fully human monoclonal antibody 1D9 that targets the immunodominant *Staphylococcal* antigen A (IsaA) of *S*. *aureus* and a near-infrared dye NIR680 [167]. Infected mice were visualized with combined PET-CT, fluorescence, and bioluminescence imaging. It was demonstrated that infection could be detected efficiently and then surgically debrided (Fig. 4F). ^89^Zr-NIR680-1D9 permitted evaluation of the stability of 1D9 localization for 11 days, compared to only a few hours with ^18^F-FDG, a conventional PET-CT radiotracer. This is due to the longer half-life of ^89^Zr compared to ^18^F-FDG. This approach of fluorescence image-guided surgery enabled real-time visualization of the extent of fluorescently labeled *S. aureus*-infected tissue for improved surgical resection.

Bacteria-enabled fluorescence imaging is a promising method for cancer detection and surgical resection, as tumor-selective fluorescent bacteria enable easy determination of tumor boundaries [206]. A strategy based on *E. coli* Nissle 1917 (EcN) nitroreductase (NTR) with a polyethylene glycol (PEG) polymer coating (PC-EcN-NTR) was developed to enhance in vivo fluorescence imaging of tumors and image-guided surgery [207]. *E. coli* was genetically engineered to constitutively overexpress NTR for fluorescence probe activation. Since the reticuloendothelial system can rapidly clear EcN, surface modification of EcN-NTR was performed to produce polymer-coated EcN-NTR and improve its circulation time. PC-EcN-NTR was injected into tumor-bearing mice intravenously. Six days after colonization, the skin of the subcutaneous tumor was uncovered and sprayed with NTR-activated fluorescence probes. The tumor showed a fluorescence signal within 5 min with a 3.15-fold enhanced signal (compared to the tumor without bacteria colonization), and the fluorescence signal was maintained for at least 3 h, which is suitable for a surgical operation. In addition, fluorescence appeared at the boundary of the tumor tissue, approximately 200 μm away from the edge. Furthermore, ex vivo fluorescence imaging of the resected tumor showed a high intensity within the tumor, while no signal was observed in other organs. Similar results were obtained with the NIR probe. This PC-EcN-NTR based-strategy has great potential in guiding the surgical resection of tumors.

## Conclusions and outlook

BWCBs have been developed as detection tools for disease diagnosis and as imaging vectors for disease detection, treatment efficacy monitoring, and image-guided surgery. These bacteria-based technologies have distinctive advantages compared to the current clinical gold standards and other nano- and bio-sensing technologies in development. To highlight a few, the simple and easily modifiable bacterial genome combined with the democratization of synthetic biology has opened up exciting possibilities in developing intricate, accurate, highly sensitive and specific sensing schemes that are easily tunable and adaptable for diverse applications. Bacteria carry out analyte processing and produce detectable signals, obviating the need for sample purification and evaluation using specialized laboratory equipment. Bacteria's ease of culture, storage, and use allows for wide accessibility of BWCBs. Given the degradability of purely biological BWCBs and the emergence of approaches for biocontainment, their release into the environment is expected to have a negligeable impact. Therefore, BWCBs could be considered as a sustainable detection method offering an attractive alternative to the more cumbersome and costly analytical instruments. Lastly, existing scalable bacterial culturing techniques can be leveraged for highly affordable mass production of BWCBs. Here, we highlight several challenges and opportunities that should be further explored to facilitate the clinical translation of these bacterial-based systems.

Firstly, safety and biocontainment must be a major consideration for in vivo application. This includes the choice of bacterial strain, in vivo growth characteristics, emergence of antibiotic resistance and other evolutionary traits, and horizontal gene transfer (HGT). Many of the Generally recognized as safe (GRAS) bacterial strains are amenable to genetic engineering and have been effectively used as chassis in BWCBs development. Furthermore, the precedence of FDA-approved live vaccines (*e.g.*, Vivotif or Bacille Calmette-Guérin) can guide the development of BWCBs using attenuated pathogens. Key advances in bacterial genome engineering and synthetic biology tools provide approaches to target and edit pathogenic bacterial genomes through the introduction of mutations (attenuated bacteria) and/or artificial plasmids to alleviate safety concerns (comprehensively reviewed in [31, 208, 209]). Such methods include kill switch-like genetic programs to kill the engineered bacteria if needed [145, 210], and auxotrophic containment to survive only in predefined environment [17, 211]. The regulatory guidelines that govern the approval and use of engineered bacteria in therapeutics and diagnostics in humans are overseen by the Center for Biologics Evaluation and Research at the US FDA. The latest FDA Recommendations for Microbial Vectors used for Gene Therapy represents the general requirements for engineering bacterial strain for clinical applications and can guide the use of microbial vectors for other in vivo applications, including disease diagnosis and image-guided surgery [212–214]. The close interactions and cross-talks between the human cells, gut microbiota, and foreign microorganisms, may lead to HGT. Analysis of the human genome has shown that bacterial genes have been potentially transferred into the human genome [215, 216]. Another study showed that the integration of bacterial DNA in the human genome occurs more frequently in tumors than in normal tissue [217]. HGT is made possible in large part by the existence of mobile genetic elements, such as plasmids and transposons [215]. Given the prevalence of plasmid use in bacterial-based diagnostics, there is a potential risk of integrating bacterial genes and synthetic circuit parts into the host or the host microbiome. Nevertheless, using GRAS organisms lowers the risks of inserting toxic pathogenic genes. In addition, HGT is common in prokaryotes [218] but very rare in eukaryotes [219]. As far as the transfer to the host microbiome, it has been shown that physical proximity can have an overriding effect on HGT. Furthermore, low DNA homology between the non-native and native DNA significantly reduces the possibility of acquiring DNA fragments even if the HGT conditions are favorable [220]. Finally if HGT cannot be prevented, genetic engineering allows to redesign the genes that are expressed in the host cells and prevent their transcription or translation in other prokaryotes in the wild [221, 222].

The functional robustness of engineered bacteria over time and in changing environments is another major consideration. Genetic circuits are based on biochemical interactions within the host cells where they use host resources (*e.g.*, transcription and translation machinery) to function. Engineered bacteria often exhibit decreased growth rates since some resources are directed toward the genetic circuit. The slow growth prompts the host organism to evolve away from the burdensome circuit via homologous recombination, point mutations/deletions, or copy number reduction [223, 224]. Homologous recombination and mutations can result in the loss of circuit elements and circuit failure. Such obstacles can be prevented by constructing libraries of parts with redundant functions with sufficient sequence diversity to reduce recombination [225, 226]. Other strategies include the use of feedback loop regulating the expression intensity of the synthetic construct in response to the metabolic status of the cell [227]. In addition, synthetic DNA sponge-mediated protein sequestration can be used to mitigate the cellular burden arisen from the increased expression of sensitive regulatory proteins in the system [228]. It should also be noted that genetic circuits are subject to stochastic fluctuations or noise. In fact, molecules involved in gene regulation are expressed at a low level which can influence the functionality of genetic circuits and may fundamentally limit the accuracy of the process [229]. Noise can be suppressed using proportional, integral, and derivative (PID) based feedback controllers [230] or negative feedback regulation [231]. A fundamental systems engineering challenge arises when interconnecting different genetic parts and circuits (*e.g.*, NOT gate, AND gate). A downstream genetic element could influence an upstream element and vice versa; thus, circuit behavior could change from connecting new downstream modules to the circuit [232, 233]. For instance, if the first gate produces the inducer of the second gate, a delayed circuit response might occur due to a delay in induction (*i.e.*, expression of the required amount of protein to induce/repress promoter activity) [233]. This delay can be resolved by increasing the expression of the problematic circuit component; however, this increase in expression might lead to toxicity for bacteria. Other contributors to circuit performance variations include strain variation (*e.g.*, differences in growth rate, transcription, and translation machinery). Such strain-based variation can be quantified using a reference plasmid that reports promoter activity by normalizing the measurements to a constitutive promoter standard treated at the same conditions [234, 235].

**Fig. 5:**
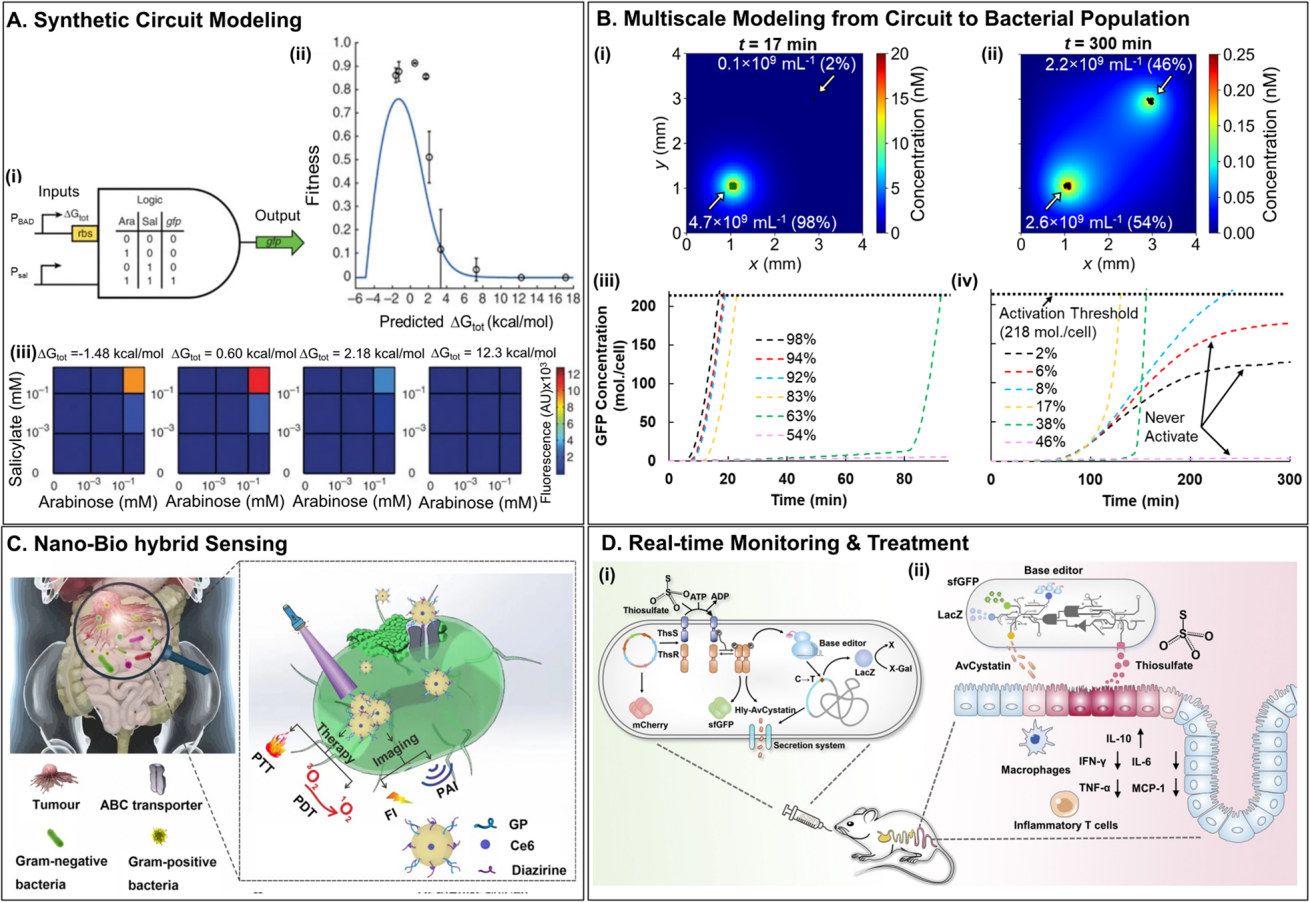
**A** Rational design of an AND gate. (i) An AND-gate genetic circuit produces the *gfp* reporter output only when both *P*_*BAD*_ and* P*_*sal*_ are induced. (ii) A quantitative model for the design of ribosomal binding sites (RBS) predicts the accuracy of circuits logic (fitness) as a function of ΔG_tot_ of the *P*_BAD_ promoter’s RBS sequence (blue line). The experimental data (circles) are in good agreement with the model. (iii) The GFP expression level in response to combinations of arabinose (0.0, 1.3 × 10^−3^, 8.3 × 10^−2^ and 1.3 mM) and salicylate (0.0, 6.1 × 10^−4^, 3.9 × 10^−2^ and 0.62 mM) for the optimal (ΔG_tot_ = -1.48 kcal/mol) and 3 other RBS designs. Reprinted with permission from [237]. **B** Emergent behavior of engineered quorum-sensing bacteria is regulated by localized decision-making. Autoinducer signal concentration field for two sub-populations at (i) 98% and 2% and (ii) 54% and 46% ratios. Green indicates steady-state activation, and black (2%) indicates steady-state inactivation. Average intracellular GFP concentration for agents of the larger (iii) and smaller (iv) populations vs. time for different population ratios. Reprinted with permission from [241]. **C** Representation of Gram-negative and Gram-positive bacterial uptake of gold nanoparticles for aggregation-enhanced imaging and antibacterial effect. Gold nanoparticle uptake enables dual mode (fluorescence imaging (FI) and photoacoustic imaging (PAI)) and combinatorial (photothermal therapy (PTT) and photodynamic therapy (PDT)) therapy. Reprinted with permission from [157]. **D** Schematic diagram of intelligent, responsive bacteria for diagnosis and therapy (i-ROBOT) system for diagnosis, recording, and treatment of colitis. (i) Schematic of the engineered components and pathways. (ii) i-ROBOT senses the colitis biomarker thiosulfate, produces real-time (fluorescence) signal, and records the detection event (genomic molecular recording and colorimetric output). Thiosulfate also regulates the drug-protein AvCystatin release to treat mouse colitis. Reprinted with permission from [23]

BWCBs face other hurdles to functional robustness in complex and heterogenous clinical samples or in vivo. To address detection robustness, biosensor designs have transitioned from single biomarker detection to integrating several inputs [9, 14, 17]. To enhance detection accuracy in the complex settings with low signal-to-noise ratio, synthetic genetic circuits capable of amplifying signals [9, 28, 236] are developed. For example, as described in Sect. "Biosensing of glucose level", multiplexed signal processing (*i.e.,* AND, NAND and NOR gates) improved the performance of BWCBs in human clinical samples [9]. Computational modeling of these sophisticated genetic circuits to tune their activity for increased sensitivity and high signal-to-noise ratio would be crucial to streamlining the design-build-test-learn cycle of synthetic biology [225, 237, 238] (Fig. 5A). This topic has been comprehensively reviewed elsewhere [223, 239]. Lastly, recent works have also investigated how spatiotemporal heterogeneity in input signals or BWCBs distribution would affect their function. Presently, design of synthetic genetic circuits tailored toward specific performance characteristics is an iterative process that relies on experimental screening of spatially homogeneous engineered cell suspensions. However, BWCBs distribute heterogeneously in complex in vivo environments, which may alter circuit performance. To address this challenge, multiscale predictive models that describe the spatiotemporal dynamics of emergent behaviors in BWCBs must be utilized to inform synthetic gene circuit design [240, 241] and support experimental efforts (Fig. 5B). Other strategies and tools from synthetic biology have been investigated to optimize biosensors’ performance. Such strategies (comprehensively reviewed in [31]) include improving specificity, lowering the limit of detection, increasing output dynamic range and reducing leakiness.

BWCBs’ functions can be augmented by interfacing bacteria with nano-particles and devices [138, 139, 154, 157] (Fig. 5C). Reproducibility and scalability in biomanufacturing these bio-hybrid systems are critical to their functions and future implementation. In addition, the effect of surface-associated or intracellular nanoparticles on bacteria growth, motility, and host interactions is rarely explored. Such considerations must be taken into account in designing biohybrid BWCBs toward achieving maximal nanoparticle load with minimal effect on motility and other application-specific critical functions to ensure robust and reproducible performance [242].

Localization of bacteria to the disease site is another crucial challenge that must be considered. Highly selective colonization of the targeted site and effective distribution within the target tissue are crucial for sensitive detection and high signal-to-noise ratio imaging. Bacteria's intrinsic ability to selectively colonize cancerous tissue can be advantageous for developing cancer-specific detection and diagnosis tools; however, effective bacterial penetration in extracellular matrix (ECM)-rich solid tumors and metastatic nodules need further investigation, as prior studies have shown that high ECM content limits bacteria penetration [70, 243]. Directed propulsion of bacteria-based systems using chemical [244] or physical stimuli such as magnetic fields [245, 246] could potentially help to alleviate this challenge. Furthermore, localizing bacteria to the sites of diseases other than cancer remains an active area of research. For instance, as discussed earlier in this review, different bacterial strains have been screened for tropism for myocardial infarction [153]. Another strategy for effective localization of the bacteria in the target environment consisted of using a magnetic hydrogel retentive system that could be localized by a wearable magnet. This device enabled spatial localization and temporal retention for long-term disease diagnosis in the intestine [37].

Another major design consideration for engineering BWCBs is the use of colonizing or non-colonizing strain. In fact, colonizing bacteria enable long-term monitoring [247]; however, they alter the host microbiota and could result in undesired side effects. While non-colonizing strains are capable of sensing relevant biomarkers during their transitory stay [247]; however, they require frequent dosing as their residence time in the body is short [248]. Ultimately, the choice of colonizing or non-colonizing strain depends on the type of application. For cancer detection and imaging, bacteria with tumor-specific colonization ability such as *E. coli* [115, 116], *Salmonella* [67, 117, 118], *Clostridium* [119, 120], and *Bifidobacterium* [121, 122] are selected. On the other hand, bacteria such as *E. coli* with short-term colonization in human after oral administration, allowed the detection of gut inflammation [13]. 

Another important consideration for the practical use of BWCBs is their shelf life. A promising approach is lyophilization, where bacterial cells are freeze-dried to allow long-term storage and successful revival and maintenance of functional performance after months and even years of storage [249, 250]. For example, *Photobacterium leiognathid* bacteria, used for bioluminescence-based UTI detection [20], were lyophilized and stored at room temperature for 3 months. Upon reconstitution, *P. leiognathi* bioluminescence intensity was comparable to that of fresh culture. However, it should be noted that certain bacteria might not survive the lyophilization process, resulting in reduction in viable count or rapid death after reconstitution [251]. Using spore-forming microorganisms as vehicles for BWCBs can be considered as an alternate approach, obviating the need for long-term storage strategies. For example, *C. novyi-*NT spores were labeled with iron-oxide nanoparticles for MRI of liver tumors [139]. These studies demonstrated that the performance of the spore-based sensors did not change compared with the labeled vegetative stage of *C. novyi-NT*. Although sporulated organisms can address the shelf-life and storage limitations, the incubation and response times will be extended due to the additional germination step requirement.

BWCBs, as self-replicating and regenerative sensors, offer an unprecedented opportunity for long-term or continuous monitoring. This capability enables timely detection of disease progression or flare-ups. Current nano- and bio-sensors are often triggered by the unidirectional binding of analytes; thus, they are intended for single use and cannot be reset easily. Synthetic circuits in BWCBs may be engineered to reset to accommodate continuous monitoring. Recent works using the CRISPR-based genome editing technology have begun to show the potential for continuous monitoring [23] (Fig. 5D) but advances in managing colonization resistance [252], real-time detection, and data transmission are needed. Other CRISPR/Cas based memory devices may be harnessed for continuous monitoring in biomedical applications. A “biological tape recorder” called temporal recording in arrays by CRISPR expansion (TRACE) [253] was developed to record intracellular DNA production triggered by biological signals. TRACE could be used to record metabolite fluctuations, gene expression changes, and lineage-associated information across cell populations. Another memory system, CRISPR-mediated analog multi-event recording apparatus (CAMERA) [254], enables durable, analog recording of stimuli and cell states. Such systems are sensitive and multiplexable [253, 254], and thus may be useful for continuous monitoring of diseases.

Implementation of BWCBs in wearable devices can provide an opportunity for detecting biomarkers from body fluids, such as sweat, for non-invasive monitoring. Such devices must allow diffusion of nutrient and oxygen to sustain the BWCBs while meeting the biocontainment and wearability requirements. Early work in this area is connected to the emerging field engineered living material and is focused on developing the requisite biomaterial matrix. Encouraging early results have reported sensing function using synthetic analytes [255, 256].

BWCB are also poised to significantly aid in expounding our knowledge of the complex bidirectional communication network between the central nervous system and the intestine, termed the gut-brain axis [257, 258]. For instance, biosensor detection of gut metabolites offers the potential to quantify the microbiome’s contributions to depression [259, 260]. BWCB also offers the opportunity to study, diagnose, and treat disorders of gut-brain interaction (formerly known as functional gastrointestinal disorders). For example, BWCBs that detect the neurotransmitter serotonin have been used for IBD detection [261].

Bacteria-specific imaging using different imaging modalities could provide continuous and quantitative information on the anatomical location of bacterial colonization and density. However, when contrast agents (e.g., radiotracers for PET) are needed, specificity to the BWCBs is critical to distinguish between the BWCBs and pathogenic bacteria or the normal microbiota and avoid false-positives [262]. It should also be noted that when reporter genes are used for bacteria-based imaging, the expression of reporter genes may diminish over time (*e.g*., due to the lack of exogenous substrate for bioluminescent bacteria), resulting in decreased signal intensity [114]. Lastly, fluorescence imaging is affected by the background autofluorescence which can reduce the sensitivity of the method [136]. Strategies and factors may be implemented to correct for autofluorescence [263]. Alternatively, other optical imaging modalities (*e.g.,* bioluminescence or photoacoustic imaging) may be implemented to circumvent this challenge.

As is the case with the developmental stage of all theranostic technologies, many of the advances in the development of BWCBs have been demonstrated in vitro, in preclinical animal models, or in clinical samples. The translation of these technologies into clinical settings has yet to be comprehensively validated. Pre-clinical evaluation of bacteria-based diagnostics is particularly challenging due to the fundamentally different nature of human and laboratory animal microbiomes and vast differences in host–pathogen interactions. In vitro models that meaningfully recapitulate human physiology and host-bacteria interactions are critical to preclinical validation of BWCBs and bridging the chasm between the preclinical and clinical success. Understanding host-bacterial interactions and the forces that shape host-associated microbial communities would help realize “ideal” biomimetic models. These models would improve the study and allow well-controlled and repeatable conditions for evaluating the BWCBs. Despite the significant progress in the complexity of in vitro models, many challenges remain. For instance, in vitro gut models cannot simultaneously recapitulate all the key conditions found in the gut or reproduce the complex microbiome-host interactions [264, 265]. Recent advances have identified the key features that must be recapitulated in vitro to attain outcomes that are predictive of the in vivo behavior [264]. Yet, available models, such as organoids [266, 267], 3D intestinal scaffolds [268], and gut-on-chip [269], have only been able to integrate a subset of those parameters [264, 265]. Thus, there is still a significant opportunity to improve in vitro models.

In conclusion, bacteria-whole-cell biosensors are uniquely advantageous as rapid, sensitive, accurate, sustainable, and cost-effective detection and diagnosis tools. There remain many exciting opportunities to build upon marvelous advances in the field to address the outstanding challenges of these technologies. One such promising opportunity is continuous and real-time monitoring. With the continuous progress in synthetic and molecular biology and bioengineering, this field is poised for rapid growth and advancement toward clinical translation.

## Data Availability

Data sharing is not applicable to this article as no datasets were generated or analyzed during the current study.

## Abbreviations

[^18^F]FEAU: ^18^F-2′-Fluoro-2′deoxy-1-β-D-arabinofuranosyl-5-ethyl-uracil
[^124^I]FIAU: ^124^I-2’-fluoro-1-β-D-arabino-furanosyl-5-iodo-uracil
[^125^I]FIAU: ^125^I-2’-fluoro-1-β-D-arabino-furanosyl-5-iodo-uracil
[^18^F]FLT: 3′-Deoxy-3′-[^18^F]fluorothymidine
AHL: N-acyl homoserine lactone
AI-1: N-3-oxohexanoyl-l-homoserine lactone
AI-2: Autoinducer-2
ARG: Acoustic Reporter Gene
ATC: Anhydrotetracycline
BURST: Burst Ultrasound Reconstructed with Signal Templates
BWCB: Bacterial whole-Cell Biosensor
Ce6: Chlorin e6
CFP: Cyan Fluorescent Protein
CFU: Colony Forming Unit
CPS: Capsular Polysaccharide
CT: Computed Tomography
CUBET: Cellphone-based UTI Bioluminescence Extinction Technology
Doxy: Doxycycline
DSS: Dextran Sodium Sulfate
FI: Fluorescence Imaging
fim: Fimbriae
GalCCP: *N*-Cyclopropenyl galactosaminyl carbamate
GDH: Glucose dehydrogenase
GFP: Green Fluorescent Protein
GP: Glucose Polymer
hFIB: Human fibrinogen
HGT: Horizontal Gene Transfer
HSV1-TK: Simplex virus type 1 thymidine kinase
IBD: Inflammatory Bowel Disease
ICG: Indocyanine green
IGS: Image-Guided Surgery
IMBED: Ingestible Micro-Bio-Electronic Device
INP: Ice-Nucleation Protein
IRL: Inverted Repeat Left
iROBOT: Intelligent responsive bacteria for diagnosis and therapy
IRR: Inverted Repeat Right
IsaA: Immunodominant *Staphylococcal* antigen A
KDO: 2-Keto-3-deoxy-D-mannooctanoic acid
KDOAz: 8-Azido-8-deoxy-KDO
LAT: Latex agglutination test
LPS: Lipopolysaccharide
Lux: Luciferase
MCAs: Microbubble Contrast Agents
MDP: Maltodextrin-based imaging probe
MDP1: Maltodextrin-based imaging probe 1
MDP2: Maltodextrin-based imaging probe 2
MRI: Magnetic Resonance Imaging
MRSA: Methicillin-Resistant *Staphylococcus aureus*
MSOT: Multispectral Optoacoustic Tomography
NIR: Near-Infrared
NTR: Nitroreductase
PBS: Phosphate-Buffered Saline
PDAC: Pancreatic Ductal Adenocarcinoma
pDAC: Polydopamine nanoparticles
PEG: Polyethylene glycol
PET: Positron emission tomography
PGN: Peptidoglycan
PID: Proportional, Integral, and Derivative
PK/PD: Pharmacokinetic/Pharmacodynamic
ppGpp: 5′-Diphosphate-3′-diphosphate
RFP: Red Fluorescent Protein
SA: Synthetic adhesin
SICE: Stress-Inducible Controlled Expression
SPECT: Single Photon Emission Computed Tomography
TK: Thymidine Kinase
TuBETUr: Tube Bioluminescence Extinction Technology Urine
UTI: Urinary Tract Infection
YFP: Yellow Fluorescent Protein
